# Prognostic model for multiple myeloma progression integrating gene expression and clinical features

**DOI:** 10.1093/gigascience/giz153

**Published:** 2019-12-30

**Authors:** Chen Sun, Hongyang Li, Ryan E Mills, Yuanfang Guan

**Affiliations:** Department of Computational Medicine and Bioinformatics, University of Michigan, 100 Washtenaw Avenue, Ann Arbor, MI 48109, USA; Department of Computational Medicine and Bioinformatics, University of Michigan, 100 Washtenaw Avenue, Ann Arbor, MI 48109, USA; Department of Computational Medicine and Bioinformatics, University of Michigan, 100 Washtenaw Avenue, Ann Arbor, MI 48109, USA; Department of Human Genetics, University of Michigan, 1241 East Catherine Street, Ann Arbor, MI 48109, USA; Department of Computational Medicine and Bioinformatics, University of Michigan, 100 Washtenaw Avenue, Ann Arbor, MI 48109, USA; Department of Internal Medicine, Nephrology Division, University of Michigan, 1150 West Medical Center Drive, Ann Arbor, MI 48109, USA

**Keywords:** multiple myeloma, prognostic model, survival analysis, GuanRank, gene signature

## Abstract

**Background:**

Multiple myeloma (MM) is a hematological cancer caused by abnormal accumulation of monoclonal plasma cells in bone marrow. With the increase in treatment options, risk-adapted therapy is becoming more and more important. Survival analysis is commonly applied to study progression or other events of interest and stratify the risk of patients.

**Results:**

In this study, we present the current state-of-the-art model for MM prognosis and the molecular biomarker set for stratification: the winning algorithm in the 2017 Multiple Myeloma DREAM Challenge, Sub-Challenge 3. Specifically, we built a non-parametric complete hazard ranking model to map the right-censored data into a linear space, where commonplace machine learning techniques, such as Gaussian process regression and random forests, can play their roles. Our model integrated both the gene expression profile and clinical features to predict the progression of MM. Compared with conventional models, such as Cox model and random survival forests, our model achieved higher accuracy in 3 within-cohort predictions. In addition, it showed robust predictive power in cross-cohort validations. Key molecular signatures related to MM progression were identified from our model, which may function as the core determinants of MM progression and provide important guidance for future research and clinical practice. Functional enrichment analysis and mammalian gene-gene interaction network revealed crucial biological processes and pathways involved in MM progression. The model is dockerized and publicly available at https://www.synapse.org/#!Synapse:syn11459638. Both data and reproducible code are included in the docker.

**Conclusions:**

We present the current state-of-the-art prognostic model for MM integrating gene expression and clinical features validated in an independent test set.

## Background

Multiple myeloma (MM) is a hematologic malignant neoplasm with wide clinical presentation and heterogeneous genetic background, characterized by bone marrow infiltration with clonal plasma cells [1–3]. MM is the third most common hematologic cancer in the USA, with an estimated 30,770 new diagnoses and 12,770 deaths in 2018 [4]. Since the first case of MM was reported in 1844, great progress has been made in its diagnosis, as shown in the International Myeloma Working Group Diagnostic Criteria for MM [5, 6]. Currently, risk-adapted therapy is becoming the standard of care. As survival analysis is essential for therapeutic decision making and clinical research, there is an urgent need to develop reliable and robust models for estimating the survival from massive time-to-event data.

A major challenge of analyzing time-to-event data is the censoring problem—the patient status is not fully available owing to tracking interruption or time limit of a study. In this work, we focused on the right-censored data, in which the censored patient did not have disease progression at the censoring time but his/her future status was not available. In this case, it is not advisable to use prediction models directly. Many statistical and machine learning applications have been developed to handle censored data. As the most commonly used survival prediction technique, the Cox proportional hazards model estimates the parameters with partial likelihood function by assuming a proportional hazards condition [7]. In addition to the basic Cox model, a variety of regularized Cox models have been adopted to deal with high-dimension data, such as Lasso-Cox [8], Ridge-Cox [9], and EN-Cox [10]. However, Cox models only optimize the partial maximum likelihood function of all realized events without considering the likelihood of censored patients. In addition, Cox models require multiple assumptions that may not be met in many real situations. Random survival forests [11] is another popular model that uses a forest of survival trees to extend the basic random forest method. Compared with the Cox model, it makes few assumptions and is a completely data-driven model, but it also ignores the information from early-censored patients. Furthermore, as a tree-based model, it prefers to split the continuous variables into categorical variables.

In 2017, the Dialogue on Reverse Engineering Assessment and Method (DREAM) [12] organized the Multiple Myeloma Challenge, in which computational methods were systematically evaluated on the held-out, previously unseen benchmark datasets. DREAM Challenge, together with Multiple Myeloma Research Foundation (MMRF), UAMS, Celgene, and Dana-Farber Cancer Institute, put together the largest training and unpublished test dataset for MM in history, allowing participants to unbiasedly evaluate the algorithms in a blind dataset. In this article, we report the best-performing method by prediction accuracy in the Sub-Challenge 3 of this challenge, integrating both expression and clinical data for MM prognosis. We used a completed hazard ranking model named GuanRank [13] with Gaussian process regression (GPR) to predict the progression of MM. Our model achieved consistent better performance across different metrics than Cox and random survival forests in 3 independent cohorts. We also identified the novel, important gene signatures related to MM progression, some of which have not been reported in previous studies. Our model and results establish the new state-of-the-art in MM prognostic modeling and provide genetic insights into MM prognosis.

## Materials and Methods

### Data collection

Data used in this study were provided by the Multiple Myeloma DREAM Challenge [14]. There are 4 cohorts from different sources, GSE24080UAMS [15], HOVON65 [16], EMTAB4032 [17], and MMRF [18]. The number of patients in the cohorts is 559, 282, 147, and 636, respectively. Gene expression, clinical, and demographic data are available for all the cohorts. For the MMRF cohort, the gene expression data were generated from RNA sequencing; for the other 3 cohorts, microarray was the original method. There are 18,994, 20,514, 20,514, and 24,128 gene expression features for the cohort EMTAB4032, GSE24080UAMS, HOVON65, and MMRF, respectively. All of the genes are used as features. All the expression data were pre-processed by the challenge organizers and data providers to ensure consistency. Age and International Staging System (ISS) stages are available as clinical and demographic data. ISS is a risk-staging system based on the assessment of 2 blood tests—β_2_ microglobulin and albumin [19]. The demographic characteristics of each cohort are summarized in Table 1.

**Table 1:** Demographic characteristics of the 4 cohorts

Cohort	GSE24080UAMS	HOVON65	MMRF	EMTAB4032
No. of patients	559	282	636	147
Mean ± Standard deviation age (years)	57.18 ± 9.45	55.07 ± 7.72	64.09 ± 10.86	66.40 ± 9.92
Sex ratio (M:F)	1.52:1	1.33:1	1.46:1	1.10:1
Progression (%)	44.54	66.67	32.70	97.99
Median time to progression (days)	776.73	558.15	389.50	346.18
Death (%)	30.77	34.75	16.35	36.91
Median time to death (days)	830.62	532.23	402.50	1287.10
ISS I (%)	52.6	40.1	32.5	25.5
ISS II (%)	26.1	25.2	36.2	30.2
ISS III (%)	21.3	28.7	28.0	40.3

### Data pre-processing and GuanRank

**Figure 1:**
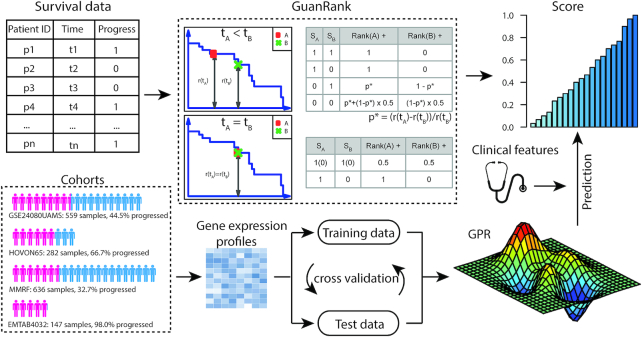
Overall workflow of the algorithm design to predict the progression of patients with MM. The original survival data were first converted into complete ranking scores via GuanRank. Four different cohorts were used to train models for predicting MM progression based on gene expression profiles and clinical features. The GPR method was used in our final model to achieve the best prediction performance.

Our model validation scheme consists of within-cohort validation and across-cohort validation. When they were evaluated within each cohort, the data were first split 5 times for 5-fold cross-validation (5 × 5 CV). For each dataset, we imputed the missing values using the mean value across patients and quantile-normalized the expression data in order to force the values into the same distribution to eliminate batch effects. The overall workflow is shown in Fig. 1.

To leverage cutting-edge machine learning techniques, we need to transform the label of time-to-event data to a new regressible label. In particular, the original label of a patient contains 2 values: (i) the binary status and (ii) the corresponding event/censoring time. This leads to the common problem that the event/censoring time cannot be used directly by typical machine learning models. Therefore, the desired new label needs to be a single value integrating the information of both the status and the time. Here we use a complete hazard-ranking algorithm, named GuanRank [20], to rank all the patients and assign a score based on their relative hazards with a Kaplan-Meier function [21]. Fig. 1 illustrates how GuanRank works. For each pair of patients, we calculated a relative rank score for each patient through pairwise comparison. When the event time of patient A is earlier than that of patient B, there are 4 scenarios: (i) if both A and B are not censored, 1 would be added to the rank score of A; (ii) if only B is censored, 1 would also be added to the rank score of A; (iii) if only A is censored, *p* would be added to the rank score of A, and 1 − *p* would be added to the rank score of B. Here *p* is a conditional probability that a future event happens before A reaches the time point of B, which can be calculated using the Kaplan-Meier survival function:

\begin{equation*} p = \frac{{r\left( {{t_A}} \right)\ - \ r\left( {{t_B}} \right)}}{{r\left( {{t_A}} \right)}}, \end{equation*}

where *r*(*t*) is the proportion of the patients that are still alive at time *t*; (iv) if both A and B are censored, *p* + (1 − *p*)/2 would be added to the rank score of A and (1 − *p*)/2 would be added to the rank score of B. When the event time of patient A is the same as that of patient B, there are 2 scenarios: (v) if both A and B are censored or neither are censored, 0.5 would be added to the rank score of A and B, respectively; (vi) if only A or B is censored, 1 would be added to the rank score of the uncensored patient. In the final model, as a summary of the above cases, the rank score of A is given by:

If *S_A_* = 1,

\begin{equation*} \mathop \sum \nolimits_{\forall B:{t_B} > {t_A}} 1 + \mathop \sum \nolimits_{\forall B:{t_B} \le {t_A},\ \ \ {S_B} = \ 0} \frac{{r\left( {{t_A}} \right)}}{{r\left( {{t_B}} \right)}} + \mathop \sum \nolimits_{\forall B:\ {t_B} = {t_A}\ ,\ \ \ {S_B} = \ 1} 0.5. \end{equation*}

If *S_A_* = 0,

\begin{eqnarray*}
&& \mathop \sum \nolimits_{\forall B:{t_B} \ge {t_A},\ \ \ {S_B} = \ 0} \left[ {1 - \frac{{0.5r\left( {{t_B}} \right)}}{{r\left( {{t_A}} \right)}}} \right] + \mathop \sum \nolimits_{\forall B:{t_B} \ge {t_A},\ \ \ {S_B} = \ 1} \left[ {1 - \frac{{r\left( {{t_B}} \right)}}{{r\left( {{t_A}} \right)}}} \right] \nonumber \\ && \quad + \mathop \sum \nolimits_{\forall B:{t_B} < {t_A},\ \ \ {S_B} = \ 0} \ \frac{{0.5r\left( {{t_A}} \right)}}{{r\left( {{t_B}} \right)}}. \end{eqnarray*}

After completing all pairwise comparison between all the patients, the relative ranking would be normalized into the range 0–1 with the following function:

\begin{equation*} \mathrm{normalized}\ \mathrm{rank}\left( A \right)\ = \frac{{\mathrm{rank}\left( A \right)\ - \ \mathrm{rank}\left( {\mathrm{min}} \right)}}{{\mathrm{rank}\left( {\mathrm{max}} \right) - \mathrm{rank}\left( {\mathrm{min}} \right)}}, \end{equation*}

where rank(*A*) is the original ranking of A, rank(min) is the minimum ranking in the cohort, and rank(max) is the maximum ranking in the cohort. Then we can use this ranking score as the target for different machine learning algorithms, such as GPR and random forest.

### Model comparison and evaluation

Four survival prediction models were compared in this study: (i) combination of GuanRank and GPR [22], (ii) GPR direct regression on progression-free survival, (iii) Lasso-Cox proportional hazards model, and (iv) random survival forests. Their performances were evaluated by 2 metrics, integrated area under the receiving operator characteristic (ROC) curve (AUC) [23] and the C-index [24]. Integrated AUC measures time-dependent concordance with the weights derived from the survival time distribution. For continuous predictions, a cut-off can be used to binarize the predictions and calculate 1 point in the ROC curve. The cut-off for continuous predictions gradually increases from 0 to 1 to obtain the ROC curve and corresponding AUC. The time-dependent AUC was first calculated at 14, 16, 18, 20, and 22 months using the weights from the Kaplan-Meier estimator of the censoring distribution [25]. Then the integrated AUC was calculated from time-dependent AUC using the weights from 2 × *S*(*t*) × *f*(*t*), where *S*(*t*) denotes the survival function and *f*(*t*) denotes the marginal density of the survival time *T_i_* as described by Heagerty et al. [23]. The C-index estimates the probability that a randomly selected patient who has experienced an event has a higher risk score than a patient who has not experienced the event [26]. Both metrics are the measures of the goodness of fit for the survival model.

### Stacking expression and clinical features

Although we have the whole gene expression profile (GEP) for the model, it was demonstrated that the GEP-alone signature has limited power to predict complete response in MM [27]. Here we combine the therapy-specific features including age and ISS. For these 2 features, we built a separate linear regression model to predict the outcomes and then stacked the results with GEP-based prediction. After trying different proportions, 50% for each model had the best performance.

### Cross-cohort prediction

To validate the robustness of the model, we predicted the MM progression across 4 cohorts. For each experiment, 2 cohorts were selected, one as the training cohort and the other as the test cohort. We used the same model and parameters as the within-cohort experiment and stacked the GEP and clinical features. Finally we had 12 different pairs of outcomes and also evaluated them with integrated AUC and C-index.

### Progression-related genes

To identify the risk genes, we built a random forest regression model based on the expression data with GuanRank hazard score as target value. Then the feature importance values for all the genes were extracted and sorted. Using the proportions of each cohort among the 4 cohorts (number of patients in 1 cohort/total number of patients in 4 cohorts) as the weights, we calculated the combined importance value for each gene. After sorting, we selected 342 genes with importance value >0.01.

To evaluate the functional properties of the gene set, we performed GO enrichment analysis with DAVID v6.8 [28]. False discovery rate correction was performed using the Benjamini-Hochberg method, and GO biological processes with Q-value <0.01 and fold enrichment >2 were considered significantly enriched. To better understand the molecular basis of MM, we put the genes into a mammalian functional network [29] context, which was constructed on the basis of a Bayesian integration of diverse genetic and functional genomic data, including protein-protein interactions, homologous functional interactome, phenotype and disease, expression and phylogenetic profiles. We then used the Girvan-Newman fast greedy algorithm [30] to perform community clustering in the network and found the enrichment function for each cluster.

## Results

In this study, we first compared the performances of MM progression prediction from GEPs between our GuanRank-GPR framework and 3 other models. By converting the original survival status into a complete ranking score, our model showed higher accuracy than the conventional survival prediction models. After integrating the clinical features, the model significantly achieved better performance. It also showed robust predictive power in cross-cohort predictions. Furthermore, we found a set of gene signatures that are important in predicting MM progression. The key biological processes and pathways associated with these genes were identified through functional enrichment analysis and gene-gene interaction network.

### GuanRank-GPR framework improves the prediction of MM progression

A major challenge in survival analysis is the incompleteness of the time-to-event data. Many efforts have been made to address this problem, including Cox regression and random survival forests. However, both of these models ignore the early-censored patient information. During the maximum likelihood (in Cox) or the random forest calculation, cases comparing an early-censored point with a late-censored or uncensored point are thrown out because of uncertainty of the relationship between the 2 points. However, let us imagine a case in which the patient was censored at 1 day (i.e., observed to be alive at Day 1), versus a patient that is censored at 10 years. Obviously, the 2 points provide important information that we could make use of. As the number of censored example goes up, we lose more information. In an extreme case where all patients are censored, we learn nothing from Cox and survival random forest.

To address this challenge, we developed the complete hazard-ranking framework, GuanRank, to estimate the relative rankings of censored patients. GuanRank differs from the traditional Cox model or random survival forests on 2 aspects: first, it gives a probability ranking of 2 individuals; even the individual with a shorter observation time is censored. In other survival models, when an early time point individual A is censored, the comparison of this individual against any of the later time point individuals B is inconclusive (Fig. 1) because we are not able to tell the status of A when it reaches the time point of B. In this case, in the maximal likelihood function used by the Cox model, this pair of A and B is discarded. However, in GuanRank, although no decisive conclusion can be made for A and B, we give a probabilistic estimation of the relationship between A and B. The intuition, as described above, is that a patient that is censored at 1 day has a higher risk at baseline than a patient that is censored at 10 years, since the former patient can die at any time in between. Effectively, we increase the sample sizes by integrating the early-censored points. Second, unlike Cox and random survival forest with fixed base learners, in GuanRank we transform the censored data problem into a standard regression problem, thus allowing us to have a much wider spectrum for base learners and making us more likely to find the most suitable one. Because GPR is particularly suitable for multi-cohort and cross-cohort modeling owing due to its local regression nature [31], with GuanRank, we were able to take advantage of GPR to significantly boost performance.

**Figure 2:**
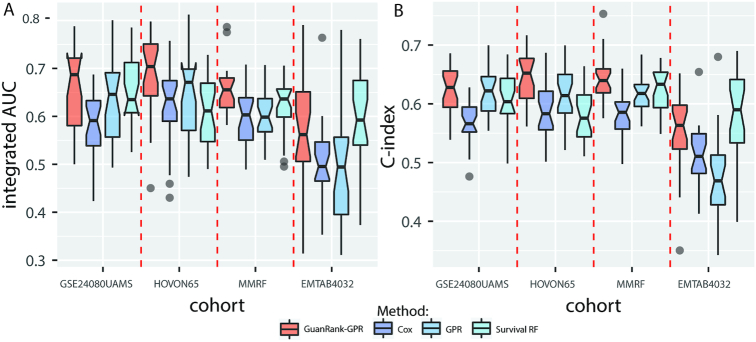
Performance comparison of different survival analysis models in 4 cohorts. In the GPR model, we directly use the progression status as the desired output to train, whereas in the GuanRank-GPR model we convert the progression status into a complete ranking score before training. The GuanRank-GPR model (red) consistently performed best in 3 cohorts (GSE24080UAMS, HOVON65, and MMRF) and second best in the EMTAB4032 cohort, when evaluated by both **(A**) integrated AUC and **(B**) C-index. GPR, Gaussian process regression. Cox, Cox proportional hazards model. RF, random forest.

To evaluate the performance of different models, we performed 5 × 5-fold cross-validation experiments within each cohort. The integrated AUC and C-index evaluation results are shown in Fig. 2. We first directly used the binary censored status as prediction target to train a GPR model. GPR is a type of Bayesian non-parametric method, and it can model complex systems while handling uncertainty in a principled manner. Fig. 2 shows that the GPR-only model performed better than Cox and random survival forests in 2 cohorts. It should be noted that values >0.5 indicate that the model is better than predicting an outcome randomly. To further consider the early-censored patient information, we calculated the continuous GuanRank scores as the prediction targets instead of the binary censored status and re-trained the GPR model (hereafter referred to as GuanRank-GPR). The GuanRank-GPR model performed best in 3 (GSE24080UAMS, HOVON65, and MMRF) of the 4 cohorts. In the EMTAB4032 cohort, our model performed slightly worse than survival random forests. In fact, most patients (143 [97%]) in this cohort experienced disease progression during the longitudinal observation period, while the progression rates (number of progressed patients/number of total patients) in the other cohort were only 45%, 67%, and 33%.

### Integrating gene expression profile and clinical data improves the performance

**Figure 3:**
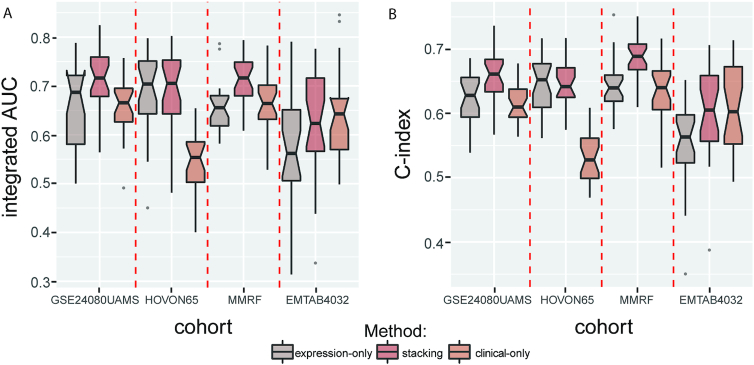
Integrating gene expression profiles and clinical features improves the prediction performance. Two types of models were built using either gene expression data or clinical data as features. After stacking the predictions from gene expression data and clinical data, the performance consistently increased in all cohorts when evaluated by **(A**) integrated AUC and **(B**) C-index, except for the integrated AUC of EMTAB4032 and the C-index of HOVON65. expression-only, models using expression data only as input features clinical-only, models using clinical data only as input features stacking, models using both expression and clinical data as input features

The GEP and clinical data are 2 types of features. They capture different aspects of information, and integrating them into our model further improves prediction performance. We combined the prediction from the clinical features, age, and ISS. The integrated AUC and C-index of the expression-only model and stacking model are shown in Fig. 3. The stacking model performed better than the expression-only model in all 4 cohorts when evaluated with integrated AUC. For the C-index metric, there was only 1 cohort for which the stacking model's performance was slightly worse. The results indicated that the prediction would be more accurate with more relative information.

### GuanRank-GPR model displays robust cross-cohort performance

**Figure 4:**
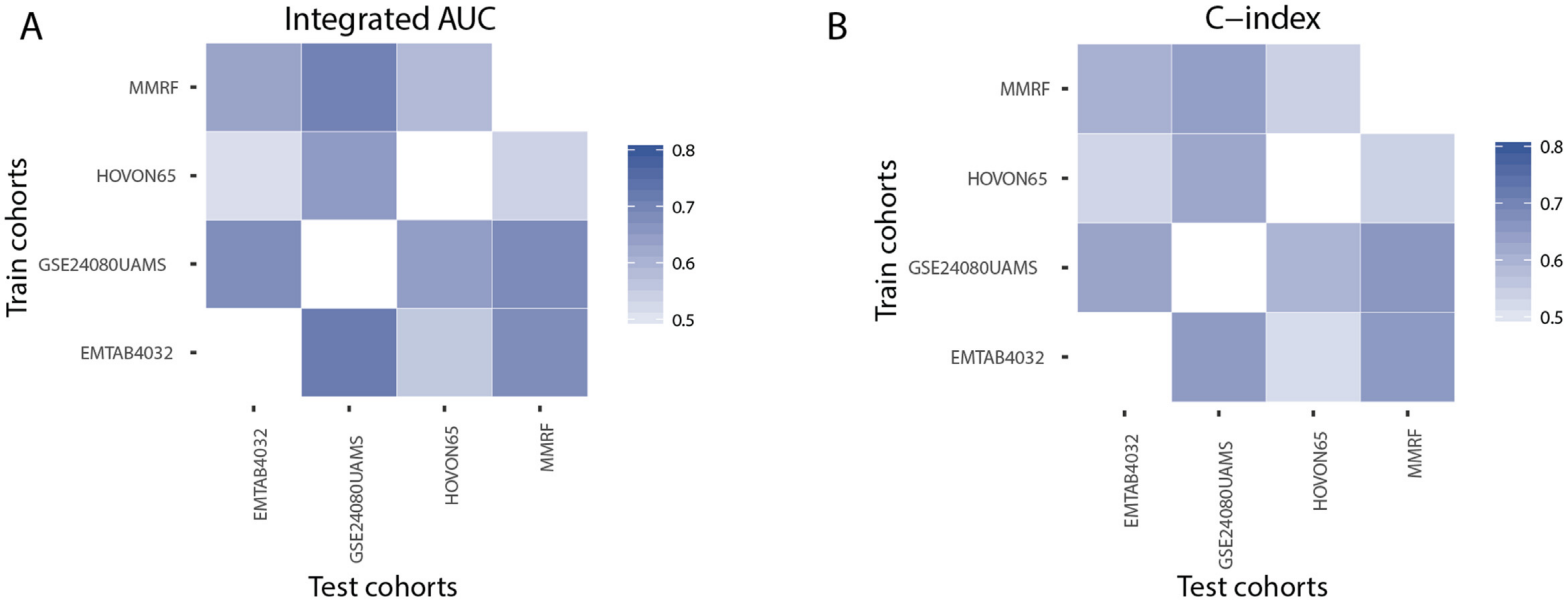
Cross-cohort evaluation of the GuanRank-GPR model. To test the robustness of our model in a cross-cohort fashion, we train our GuanRank-GPR model on one cohort and make predictions on another cohort. Each row represents the training cohort and each column represents the test cohort. There are no data in the diagonal of the matrices, which represents the self-prediction. The predictions were evaluated using **(A**) integrated AUC and **(B**) C-index.

It is difficult to predict the progression of a new patient with the information from different cohorts owing to the cohort and batch effects. A cohort is a group of people who share a common characteristic or experience within a defined period. If we just focus on 1 cohort, we cannot get the whole landscape. Therefore, cross-cohort robust models are needed. The GuanRank framework can take advantage of the information from the censored patients. It exhibits more robustness than the conventional models when the cohorts contain a certain amount of censored data. Here, we systematically evaluate the performance of our GuanRank-GPR model in a cross-cohort fashion: for each pair of cohorts, we trained our model on one cohort and validated the performance on the other. Fig. 4 illustrates the evaluation of the results of 12 training-test pairs with integrated AUC and C-index. All the values are >0.5, showing that the model is robust to cohort bias.

### Gene determinants of MM progression prediction are extracted from feature importance analysis

**Figure 5:**
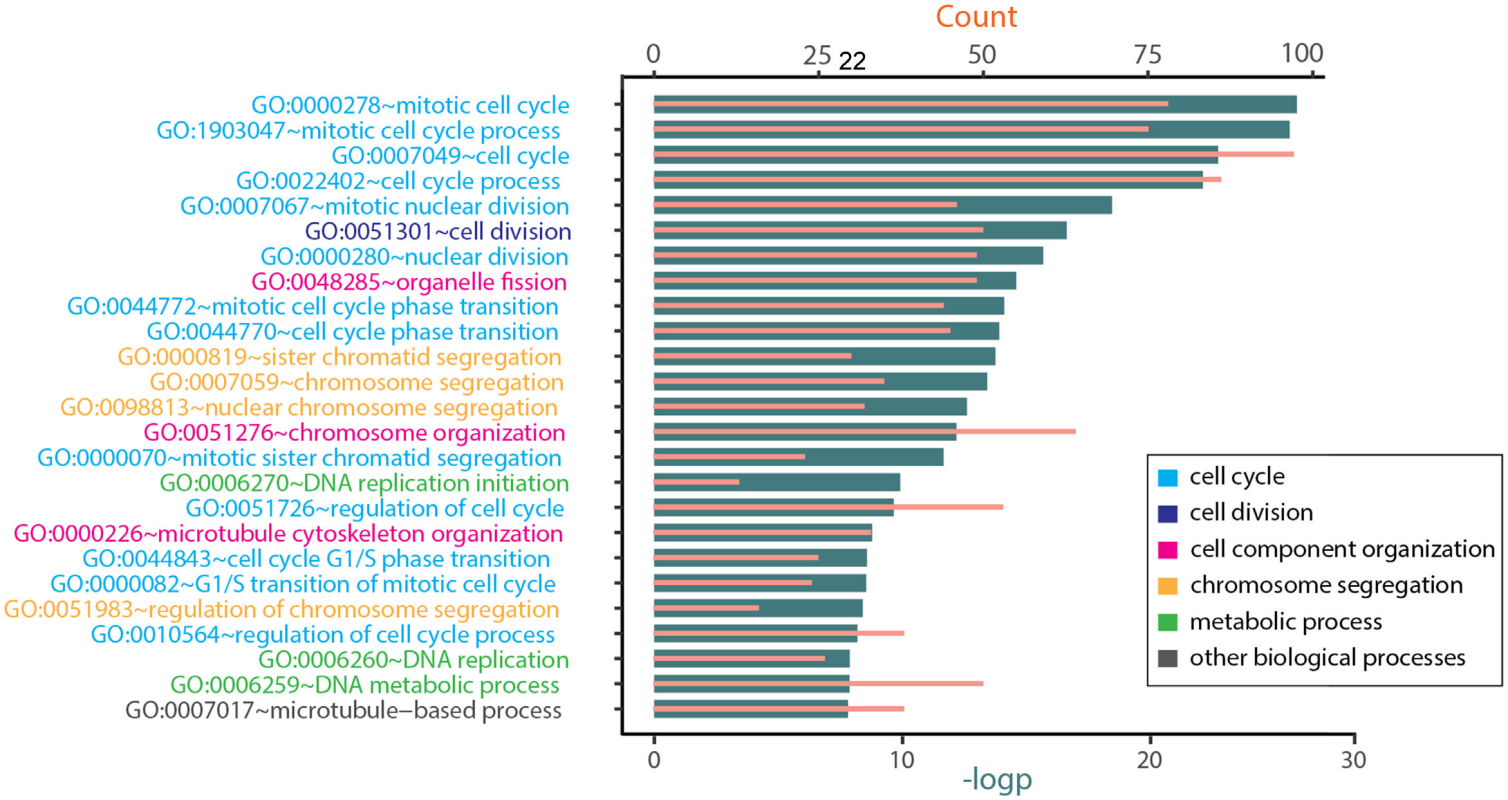
GO enrichment analysis of important genes in predicting MM progression. The top 25 significant GO biological processes were enriched from 342 key genes related to MM progression. In the list, color indicates biological process class. Most biological processes are related to the cell cycle. In the bar graph, the dark bars are the negative logarithmic transformation of the *P*-value, and the light bars are the counts of genes.

A key set of 342 gene determinants related to MM progression were identified on the basis of the feature importance from random forests regression. To investigate the functional pathways associated with these genes, we performed functional enrichment analysis. After filtering with Q-value and fold enrichment, we found 69 significantly enriched GO biological processes (Supplementary Fig. S1; Fig. 5 shows the top 25 processes). Most processes are related to the cell cycle or chromosomal instability. The cell cycle is a process by which cells progress, divide, and reproduce themselves. Proper cell cycle progression is regulated by cell cycle proteins and checkpoint pathways. However, deregulation of cell cycle progression is one of the key hallmarks of cancer [32]. Chromosomal instability is another characteristic property of cancer cells, where chromosomes are not stable as they are in normal cells [33].

**Figure 6:**
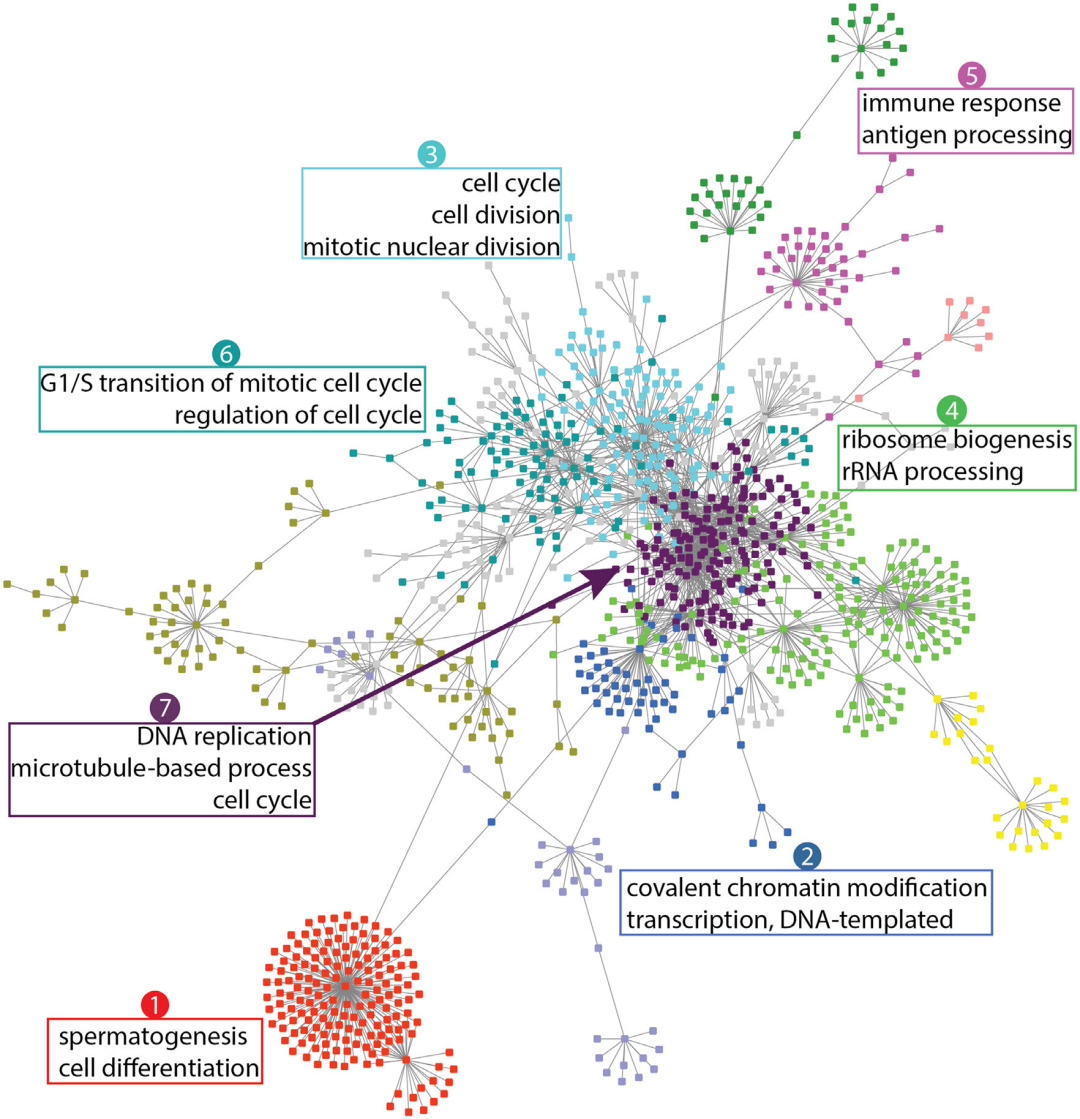
Functional clusters in the MM progression gene network. The gene clusters are shown in different colors and visualized using a mammalian gene-gene interaction network. The shared biological processes of selected clusters are labeled.

To better understand the molecular basis of the MM, we studied the gene interactions and the shared biological processes under a mammalian gene function network (Fig. 6). We clustered the functionally related genes; different colors represent different clusters. Three clusters (Cluster 3, 6, 7) are related to cell cycles, including cell division, nuclear division, and regulation and transition of the mitotic cell cycle. Other clusters are related to chromatin modification (Cluster 2), spermatogenesis (Cluster 1), ribosome biogenesis (Cluster 4), and the immune system (Cluster 5).

Several genes in our gene signature were reported in recent studies. *MYBL2* and *ANP32E* were identified as the top 2 important genes in our result. *MYBL2* is a gene that encodes a transcription factor with functions in checkpoint control of the G2 cell cycle phase. Heinrichs et al. considered this gene as a key tumor suppressor and believed that it plays an important role in myeloid malignant neoplasms [34]. *ANP32E* is a member of the acid nuclear protein family that has been implicated in histone acetyltransferase inhibitory activity. Walker et al. identified ANP32E as one of the prognostic important genes for myeloma in 372 patients with MM [35]. Furthermore, several genes have never been reported to be associated with MM, but they are oncogenes (e.g., *TPX2* is related to gastric cancer [36, 37] and pancreatic cancer [38]; *UBE2C* is related to prostate cancer [39] and colorectal cancer [40]). Further validations for these genes are needed. The complete gene signature is available in Supplementary Table S1.

## Discussion

With the development of machine learning techniques, survival analysis can benefit more from the efforts of state-of-the-art algorithms. Many machine learning approaches, as well as statistical models and their extensions, were developed for survival prediction. In this article, we used a non-parametric ranking method to assign a hazard score to each patient in the study. Then we built a Gaussian process regression model on the GEP with the hazard scores as the target. Our model outperformed other popular models when evaluated using both integrated AUC and C-index of the predictions. The model also showed robust predictive power in our cross-cohort validations.

There are 3 advantages of the GuanRank framework. First, it does not rely on any assumption, while the Cox model assumes a proportional hazards condition [7]. Second, it is easy to generalize the data into a standard regression problem, where many machine learning methods can be applied to the survival prediction. Third, it completely ranks the patient pairs including ealy-censored−late-uncensored pairs, which is not considered in the Cox [7] and random survival forests [11]. However, it also has several limitations. When an event happened in an unbalanced way within the cohort, e.g., 98% of patients experienced progression during the observation period in the EMTAB4032 cohort, the performance of our model is not as good as expected. Another problem is that our model focuses on the hazard ranking. It would lose the specific time information and only use the event order between a pair of patients.

After combining clinical information, the performance was improved. This indicates that a GEP alone is inadequate in predicting progression for MM, and it is helpful to add more progression-related features. Although GEP has been widely used for hazard risk prediction, it cannot reflect the whole landscape of MM progression. We need to develop a more comprehensive predictive model with an integrated genomics approach. Cytogenetic abnormality is another important marker for MM progression prediction, and it has been reported to provide prognostic information [41]. The DREAM Challenges also provided the cytogenetic data; however, they contained many missing values, and the performance decreased when these cytogenetics features were added. We believe that the model would have better predictive power when more high-quality cytogenetic features were stacked.

Specifically, we wanted to extract the most informative genes from the GEP in order to better predict the progression of patients with newly diagnosed MMs. We built a GEP-based prognostic signature with GuanRank hazard score as the target value. Part of the genes were reported to be associated with MM, and part of the genes were not but correlated with other types of cancers. A few gene signatures were published in recent years, such as EMC-92 [42], UAMS-70 [43], UAMS-80 [44], IFM-15 [45], MRC-IX [46], and HM-19 [47]. There is little overlap among these signatures, and they are also not included in our signature. It is shown that all the signatures have a cohort bias and cannot completely reflect MM progression. More comprehensive studies are needed in the future.

## Availability of Supporting Source Code and Requirements

Project name: Multiple Myeloma Survival Prediction

Project home page: https://www.synapse.org/#!Synapse:syn11459638

Includes: Data and dockerized environment for training and prediction

Operating system(s): Platform independent

Programming language: Perl, Python, and Matlab

License: GNU GPL v3.0

An archival copy of code and data is also available via the *GigaScience* database GigaDB [48]. The dockerized environment has been registered in bio.tools with the identifier biotools: Multiple_Myeloma_survival_prediction, and in SciCrunch with the identifier RRID:SCR_017651.

## Additional Files

Supplementary Figure S1. The complete list of significantly enriched GO biological processes.

Supplementary Table S1. The complete list of gene signatures.

giz153_GIGA-D-19-00353_Original_Submission

giz153_GIGA-D-19-00353_Revision_1

giz153_Response_to_Reviewer_Comments_Original_Submission

giz153_Reviewer_1_Report_Original_Submission Pavel Sumazin -- 10/24/2019 Reviewed

giz153_Supplemental_Files

## Abbreviations

AUC: area under the receiving operator characteristic curve; DFCI: Dana-Farber Cancer Institute; DREAM: Dialogue on Reverse Engineering Assessment and Method; GEP: gene expression profile; GO: gene ontology; GPR: Gaussian process regression; ISS: International Staging System; MM: multiple myeloma; MMRF: Multiple Myeloma Research Foundation; ROC: receiving operator characteristic; UAMS: University of Arkansas for Medical Sciences.

## Competing Interests

Y.G. receives personal payment from Eli Lilly and Company, Genentech Inc., F. Hoffmann-La Roche AG, and Cleerly Inc.; holds equity shares at Cleerly Inc. and Ann Arbor Algorithms Inc.; and receives research support from Merck KGaA as research contracts and Ryss Tech as unrestricted donations.

## Funding

This study was supported by research funding from NSF CAREER: On-line Service for Predicting Protein Phosphorylation Dynamics Under Unseen Perturbations (NSF-US14-PAF07599); University of Michigan O'Brien Kidney Translational Core Center NIH (5-P30-DK-081943-10); and Michigan Institute for Data Science (MIDAS) grant—Michigan Center for Single-Cell Genomic Data Analytics.

## Authors' Contributions

C.S. analyzed the data and drafted the manuscript. Y.G. contributed the original winning solution. C.S., H.L., R.E.M., and Y.G. edited the manuscript.
