# Supplementary material for: Prognostic model for multiple myeloma progression integrating gene expression and clinical features
Source: Gigascience. 2019 Dec 30;8(12):giz153. doi: 10.1093/gigascience/giz153 (PMC6936209; doi:10.1093/gigascience/giz153)
Supplement: giz153_GIGA-D-19-00353_Revision_1 [file giz153_giga-d-19-00353_revision_1.pdf]

GigaScience

Prognostic Model for Multiple Myeloma Progression Integrating Gene Expression and Clinical Features

--Manuscript Draft--

**Manuscript Number:**	GIGA-D-19-00353R1
**Full Title:**	Prognostic Model for Multiple Myeloma Progression Integrating Gene Expression and Clinical Features
**Article Type:**	Technical Note
**Funding Information:**	National Science Foundation (NSF-US14-PAF07599) — Dr. Yuanfang Guan<br>National Institutes of Health (5-P30-DK-081943-10) — Dr. Yuanfang Guan<br>Michigan Institute for Data Science (MIDAS) (Michigan Center for Single-Cell Genomic Data Analytics) — Dr. Yuanfang Guan
**Abstract:**	Background: Multiple Myeloma (MM) is a hematological cancer caused by abnormal accumulation of monoclonal plasma cells in bone marrow. With the increase in treatment options, risk-adapted therapy is becoming more and more important. Survival analysis is commonly applied to study progression or other events of interest and stratify the risk of patients.<br>Results: In this study, we present the current state-of-the-art model for MM prognosis and the molecular biomarker set for stratification: the winning algorithm in the 2017 Multiple Myeloma DREAM Challenge, Sub-Challenge 3. Specifically, we built a non-parametric complete hazard ranking model to map the right-censored data into a linear space, where commonplace machine learning techniques, such as Gaussian process regression and random forests, can play their roles. Our model integrated both the gene expression profile and clinical features to predict the progression of MM. Compared with conventional models, such as Cox model and Random Survival Forests, our model achieved higher accuracy in 3 within-cohort predictions. In addition, it showed robust predictive power in cross-cohort validations. Key molecular signatures related to MM progression were identified from our model, which may function as the core determinants of MM progression and provide important guidance for future research and clinical practice. Functional enrichment analysis and mammalian gene-gene interaction network revealed crucial biological processes and pathways involved in MM progression. The model is dockerized and publicly available at https://www.synapse.org/#!Synapse:syn11459638. Both data and reproducible code are included in the docker.<br>Conclusions: We present the current state-of-the-art prognostic model for Multiple Myeloma integrating gene expression and clinical features validated in independent test set.
**Corresponding Author:**	Yuanfang Guan<br><br>UNITED STATES
**Corresponding Author Secondary Information:**	
**Corresponding Author's Institution:**	
**Corresponding Author's Secondary Institution:**	
**First Author:**	Chen Sun
**First Author Secondary Information:**	
**Order of Authors:**	Chen Sun<br>Hongyang Li<br>Ryan E. Mills

Powered by Editorial Manager® and ProduXion Manager® from Aries Systems Corporation

	Yuanfang Guan
**Order of Authors Secondary Information:**	
**Response to Reviewers:**	Dear editor,<br><br>Thank you for your comments. We have thoroughly revised the manuscript as the instructions.<br><br>Best wishes,<br>Yuanfang Guan
**Additional Information:**	
**Question**	**Response**
Are you submitting this manuscript to a special series or article collection?	No
**Experimental design and statistics**<br><br>Full details of the experimental design and statistical methods used should be given in the Methods section, as detailed in our Minimum Standards Reporting Checklist. Information essential to interpreting the data presented should be made available in the figure legends.<br><br>Have you included all the information requested in your manuscript?	Yes
**Resources**<br><br>A description of all resources used, including antibodies, cell lines, animals and software tools, with enough information to allow them to be uniquely identified, should be included in the Methods section. Authors are strongly encouraged to cite Research Resource Identifiers (RRIDs) for antibodies, model organisms and tools, where possible.<br><br>Have you included the information requested as detailed in our Minimum Standards Reporting Checklist?	Yes
**Availability of data and materials**<br><br>All datasets and code on which the	Yes

conclusions of the paper rely must be either included in your submission or deposited in publicly available repositories (where available and ethically appropriate), referencing such data using a unique identifier in the references and in the “Availability of Data and Materials” section of your manuscript.<br><br>Have you have met the above requirement as detailed in our Minimum Standards Reporting Checklist?	

Powered by Editorial Manager® and ProduXion Manager® from Aries Systems Corporation

Manuscript

Click here to view linked References

Click here to access/download;Manuscript;Multiple_Myeloma_manuscript_Gig

Prognostic Model for Multiple Myeloma Progression Integrating Gene Expression and Clinical Features

Chen Sun[1,+], Hongyang Li[1,+], Ryan E. Mills[1,2], Yuanfang Guan[1,3,*]

1. Department of Computational Medicine and Bioinformatics, University of Michigan, Ann Arbor, MI, USA

2. Department of Human Genetics, University of Michigan, Ann Arbor, MI, USA

3. Department of Internal Medicine, Nephrology Division, University of Michigan, Ann Arbor, MI, USA

+These authors contributed equally to the work

* Corresponding author: Yuanfang Guan

Address: 2044 Palmer Commons, 100 Washtenaw Ave, Ann Arbor, MI 48109, USA

E-mail: gyuanfan@umich.edu

Phone: 734-764-0018

Email address:

Chen Sun: cnsun@umich.edu

Hongyang Li: hyangl@umich.edu

Ryan E. Mills: remills@med.umich.edu

Yuanfang Guan: gyuanfan@umich.edu

Abstract

Background: Multiple Myeloma (MM) is a hematological cancer caused by abnormal accumulation of monoclonal plasma cells in bone marrow. With the increase in treatment options, risk-adapted therapy is becoming more and more important. Survival analysis is commonly applied to study progression or other events of interest and stratify the risk of patients.

Results: In this study, we present the current state-of-the-art model for MM prognosis and the molecular biomarker set for stratification: the winning algorithm in the 2017 Multiple Myeloma DREAM Challenge, Sub-Challenge 3. Specifically, we built a non-parametric complete hazard ranking model to map the right-censored data into a linear space, where commonplace machine learning techniques, such as Gaussian process regression and random forests, can play their roles. Our model integrated both the gene expression profile and clinical features to predict the progression of MM. Compared with conventional models, such as Cox model and Random Survival Forests, our model achieved higher accuracy in 3 within-cohort predictions. In addition, it showed robust predictive power in cross-cohort validations. Key molecular signatures related to MM progression were identified from our model, which may function as the core determinants of MM progression and provide important guidance for future research and clinical practice. Functional enrichment analysis and mammalian gene-gene interaction network revealed crucial biological processes and pathways involved in MM progression. The model is dockerized and publicly available at https://www.synapse.org/#!Synapse:syn11459638. Both data and reproducible code are included in the docker.

Conclusions: We present the current state-of-the-art prognostic model for Multiple Myeloma integrating gene expression and clinical features validated in independent test set.

Keywords

Multiple Myeloma, prognostic model, survival analysis, GuanRank, gene signature

Background

Multiple myeloma (MM) is a hematologic malignancy with wide clinical presentation and heterogeneous genetic background, characterized by bone marrow infiltration with clonal plasma cells [1–3]. MM is the third most common hematologic cancer in the US with an estimated 30,770 new diagnoses and 12,770 deaths in 2018 [4]. Since the first case of MM was reported in 1844, great progress has been made in the diagnosis, as shown in the International Myeloma Working Group Diagnostic Criteria for MM [5,6]. Currently, risk-adapted therapy is becoming the standard of care. As survival analysis is essential for therapeutic decision making and clinical research, there is an urgent need to develop reliable and robust models for estimating the survival from massive time-to-event data.

A major challenge of analyzing time-to-event data is the censoring problem - the patient status is not fully available due to tracking interruption or time limit of a study. In this work, we focused on the right-censored data, in which the censored patient did not have disease progression at the censoring time but his/her future status was not available. In this case, it is not advisable to use prediction models directly. Many statistical and machine learning applications have been developed to handle censored data. As the most commonly used survival prediction technique, Cox proportional hazards model estimates the parameters with partial likelihood function by assuming a proportional hazards condition [7]. In addition to basic Cox model, a variety of regularized Cox models have been adopted to deal with high-dimension data, such as Lasso-Cox [8], Ridge-Cox [9] and EN-Cox [10]. However, Cox models only optimize the partial maximum likelihood function of all realized events without considering the likelihood of censored patients. In addition, Cox models require multiple assumptions that may not be met in many real situations. Random Survival Forests [11] is another popular model that uses a forest of survival trees to extend the basic random forest method. Compared with Cox model, it makes few assumptions and is a completely data driven model but it also ignores the information from early-censored patients. Furthermore, as a tree-based model, it prefers to split the continuous variables into categorical variables.

In 2017, the Dialogue on Reverse Engineering Assessment and Method (DREAM) [12] organized the Multiple Myeloma Challenge, in which computational methods were systematically evaluated on the held-out, previously unseen benchmark datasets. DREAM Challenge together with MMRF, UAMS, Celgene and DFCI, put together the largest training and unpublished test dataset for MM together in history, allowing participants to unbiasedly evaluate the algorithms in blind dataset. In this paper, we report the best-performing method by prediction accuracy in the Sub-Challenge 3 of this

challenge, integrating both expression and clinical data for MM prognosis. We employed a completed hazard ranking model named GuanRank [13] with Gaussian process regression (GPR) to predict the progression of MM. Our model achieved consistent better performance across different metrics than Cox and Random Survival Forests in three independent cohorts. We also identified the novel, important gene signatures related to MM progression, some of which have not been reported in previous studies. Our model and results establish the new state-of-the-art in MM prognostic modelling and provide genetic insights into MM prognosis.

Materials and Methods

Data collection

Data used in this study were provided by the Multiple Myeloma Dream Challenge [14]. There are 4 cohorts from different sources, GSE24080UAMS [15], HOVON65 [16], EMTAB4032 [17] and MMRF [18]. The number of patients in the cohorts is 559, 282, 147 and 636 respectively. Gene expression data, clinical and demographic data are available for all the cohorts. For MMRF cohort, the gene expression data were generated from RNA-seq; for other 3 cohorts, microarray was the original method. There are 18,994, 20,514, 20,514 and 24128 gene expression features for the cohort EMTAB4032, GSE24080UAMS, HOVON65 and MMRF, respectively. All of the genes are used as features. All the expression data were preprocessed by the challenge organizers and data providers in order to ensure consistency. Age and International Staging System (ISS) stages are available as clinical and demographic data. ISS is a risk staging system based on the assessment of two blood tests - beta 2-microglobulin and albumin [19]. The demographic characteristics of each cohort are summarized in Table 1.

Data preprocessing and GuanRank

Our model validation scheme consists of within-cohort validation and across-cohort validation. When it is evaluated within each cohort, the data was first split 5 times for 5-fold cross validation (5x5 CV). For each dataset, we imputed the missing values using the mean value across patients and quantile normalized the expression data in order to force the values into the same distribution to eliminate batch effects. The overall workflow is shown in Figure 1.

To leverage cutting edge machine learning techniques, we need to transform the label of time-to-event data to a new regressible label. In particular, the original label of a patient contains two values: (i) the binary status and (ii) the corresponding event/censoring time. This leads to the common problem that the event/censoring time can not be used directly by typical machine learning models. Therefore, the desired new label needs to be a single value integrating the information of both the status and the time. Here we employ a complete hazard ranking algorithm, named GuanRank [20], to rank all the patients and assign a score based on their relative hazards with Kaplan-Meier function [21]. Figure 1 illustrates how GuanRank works. For each pair of patients, we calculated a relative rank score for each patient through pairwise comparison. When the event time of patient A is earlier than that of patient B, there are four scenarios: 1) if both A and B are not censored, 1 would be added to the rank score of A; 2) if only B is censored, 1 would also be added to the rank score of A; 3) if only A is censored, p would be added to the rank score of A, and 1-p would be added to the rank score of B. Here p is a conditional probability that a future event happens before A reaches the time point of B, which can be calculated using Kaplan-Meier survival function:

$$p = \frac{r(t_A) - r(t_B)}{r(t_A)}$$

where r(t) is the proportion of the patients that are still alive at time t; 4) if both A and B are censored, p+(1-p)/2 would be added to the rank score of A and (1-p)/2 would be added to the rank score of B. When the event time of patient A is the same as that of patient B, there are two scenarios: 5) if both A and B are censored or none of them are censored, 0.5 would be added to the rank score of A and B respectively; 6) if only A or B is censored, 1 would be added to the rank score of not censored patient . In the final model, as a summary of the above cases, the rank score of A is given by:

If $S_A = 1$,

$$\sum_{\forall B: t_B > t_A} 1 + \sum_{\forall B: t_B \leq t_A,\, S_B = 0} \frac{r(t_A)}{r(t_B)} + \sum_{\forall B: t_B = t_A,\, S_B = 1} 0.5$$

If $S_A = 0$,

$$\sum_{\forall B: t_B \geq t_A,\, S_B = 0} \left(1 - \frac{0.5 r(t_B)}{r(t_A)}\right) + \sum_{\forall B: t_B \geq t_A,\, S_B = 1} \left(1 - \frac{r(t_B)}{r(t_A)}\right) + \sum_{\forall B: t_B < t_A,\, S_B = 0} \frac{0.5 r(t_A)}{r(t_B)}$$

After completing all pairwise comparison between all the patients, the relative ranking would be normalized into 0 to 1 with the following function:

$$normalized\ rank(A) = \frac{rank(A) - rank(min)}{rank(max) - rank(min)}$$

where rank(A) is the original ranking of A, rank(min) is the minimum ranking in the cohort, and rank(max) is the maximum ranking in the cohort. Then we can use this ranking score as the target for different machine learning algorithms, such as Gaussian process regression, random forest, etc.

Model comparison and evaluation

Four survival prediction models were compared in this study: 1) combination of GuanRank and Gaussian process regression (GPR) [22]; 2) GPR directly regress on progression-free survival (PFS); 3) Lasso-Cox proportional hazards model and 4) Random Survival Forests. Their performances were evaluated by two metrics. One is Integrated area under the ROC curve (AUC) [23], and the other is C-index [24]. Integrated AUC measures time-dependent concordance with the weights derived from the survival time distribution. For continuous predictions, a cutoff can be used to binarize the predictions and calculate one point in the ROC curve. The cutoff for continuous predictions gradually increases from 0 to 1 to obtain the ROC curve and corresponding AUC. The time-dependent AUC was first calculated at 14, 16, 18, 20, and 22 months using the weights from Kaplan-Meier estimator of the censoring distribution [25]. Then the integrated AUC was calculated from time-dependent AUC using the weights from 2*S(t)*f(t), where S(t) denoted the survival function and f(t) denoted the marginal density of the survival time Ti as described by Heagerty et al. [23] C-index estimates the probability that a randomly selected patient who has experienced an event has a higher risk score than a patient who has not experienced the event [26]. Both metrics are the measures of the goodness-of-fit for the survival model.

Stacking expression and clinical features

Although we have the whole gene expression profile (GEP) for the model, it was demonstrated that GEP-alone signature has limited power to predict complete response in MM.[27] Here we combine the therapy-specific features including age and ISS. For these two features, we built a separate linear regression model to predict the outcomes and then stacked the results with GEP-based prediction. After trying different proportions, 50% for each model had the best performance.

Cross-cohorts prediction

In order to validate the robustness of the model, we predicted the MM progression across 4 cohorts. For each experiment, two cohorts were selected, one as the training cohort and the other as the test cohort. We used the same model and parameters as the within-cohort experiment and stacked the GEP and clinical features. Finally we had 12 different pairs of outcomes and also evaluated them with Integrated AUC and C-index.

Progression related genes

To identify the risk genes, we built a random forest regression model based on the expression data with GuanRank hazard score as target value. Then the feature importance values for all the genes were extracted and sorted. Using the proportions of each cohort among the four cohorts (number of patients in one cohort / total number of patients in four cohorts) as the weights, we calculated the combined importance value for each gene. After sorting, we selected 342 genes whose importance values are higher than 0.01.

To evaluate the functional properties of the gene set, we performed GO enrichment analysis with DAVID v6.8 [28]. False discovery rate correction was performed using the Benjamini–Hochberg method, and GO biological processes whose Q-value < 0.01 and fold enrichment > 2 were considered significantly enriched. To better understand the molecular basis of MM, we put the genes into a mammalian functional network [29] context which was constructed based on a Bayesian integration of diverse genetic and functional genomic data, including protein-protein interactions, homologous functional interactome, phenotype and disease, expression and phylogenetic profiles. We then used the Girvan-Newman fast greedy algorithm [30] to perform community clustering in the network and found the enrichment function for each cluster.

Results

In this study, we first compared the performances of MM progression prediction from gene expression profiles between our GuanRank-GPR framework and other 3 models. By converting the original survival status into a complete ranking score, our model showed higher accuracy than the conventional survival prediction models. After integrating the clinical features, the model significantly achieved better performance. It also showed robust predictive power in cross-cohort predictions. Furthermore, we found a set of gene signatures that are important in predicting MM progression. The key biological processes and pathways associated with these genes were identified through functional enrichment analysis and gene-gene interaction network.

GuanRank-GPR framework improves the prediction of MM progression

A major challenge in survival analysis is the incompleteness of the time-to-event data. Many efforts have been made to address this problem, including Cox regression and Random Survival Forests. However, both of these models ignore the early-censored patient information. During the maximum likelihood (in Cox) or the random forest calculation, cases where an early censored point and late censored point/uncensored point is thrown out because of uncertainty of the relationship between two points. However, let us imagine a case where, the patient was censored at 1 day (i.e. observed to be alive at day 1), versus a patient that is censored at 10 years. Obviously, the two points provide important information that we could make use of. As the number of censored example goes up, we lose more information. In an extreme case where all patients are censored, we learn nothing from Cox and Survival Random Forest.

To address this challenge, we developed the complete hazard ranking framework, GuanRank, to estimate the relative rankings of censored patients. GuanRank differs from traditional Cox model or Random Survival Forests on two aspects: First, it gives a probability ranking of two individuals, even the individual with a shorter observation time is censored. In other survival models, when an early time point individual *A* is censored, the comparison of this individual against any of the later time point individuals *B* is inconclusive (Figure 1), because we are not able to tell the status of *A* when it reaches the time point of *B*. In this case, in the maximal likelihood function employed by Cox model, this pair of A and B is discarded. However, in GuanRank, though no decisive conclusion can be made for A and B, we give a probabilistic estimation of the relationship between A and B. The intuition, as described above, is that a patient that is censored at one day has a higher risk at baseline than a patient that is censored at ten years, since the former patient can die at any time in between. Effectively, we increase the sample sizes by integrating the early censored points. Secondly, unlike Cox and Random Survival Forest with fixed base learners, in GuanRank we transform the censored data problem into a standard regression problem, thus allowing us to have a much wider spectrum for base learners and make us more likely to find the most suitable one. Because Gaussian process regression (GPR) is particularly suitable for multi-cohort and cross-cohort modelling due to its local regression nature [31], with GuanRank, we were able to take in the advantage of GPR to significantly boost out performance.

To evaluate the performance of different models, we performed 5 times 5-fold cross-validation experiments within each cohort. The integrated AUC and C-index evaluation results are shown in Figure 2. We first directly used the binary censored status as prediction target to train a Gaussian process regression (GPR) model. GPR is a type of

Bayesian non-parametric method, and it can model complex systems while handling uncertainty in a principled manner. Figure 2 shows that the GPR-only model performed better than Cox and random survival forests in two cohorts. It should be noted that values over 0.5 indicates that the model is better than predicting an outcome randomly. To further consider the early-censored patient information, we calculated the continuous GuanRank scores as the prediction targets instead of the binary censored status and re-trained the GPR model (hereafter referred to as GuanRank-GPR). The GuanRank-GPR model performed best in the three (GSE24080UAMS, HOVON65 and MMRF) out of four cohorts. In the EMTAB4032 cohort, our model performed slightly worse than survival random forests. In fact, most patients (143, 98%) in this cohort progressed during the longitudinal observation period, while the progression rates (number of progressed patients / number of total patients) in the other cohort are only 44.5%, 66.7% and 32.7%.

Integrating gene expression profile and clinical data improves the performance

The gene expression profile and clinical data are two types of features. They capture different aspects of information and integrating them into our model further improves prediction performance. We combined the prediction from the clinical features, age and ISS. The integrated AUC and C-index of expression-only model and stacking model are shown in Figure 3. The stacking model performed better than expression-only model in all four cohorts when evaluated with integrated AUC. For C-index metric, there was only one cohort where stacking model's performance was slightly worse. The results indicated that the prediction would be more accurate with more relative information.

GuanRank-GPR model displays robust cross-cohort performance

It is difficult to predict the progression of a new patient with the information from different cohorts due to the cohort and batch effects. A cohort is a group of people who share a common characteristic or experience within a defined period. If we just focused on one cohort, we cannot get the whole landscape. Therefore, cross-cohort robust models are needed. The GuanRank framework can take advantage the information from the censored patients. It exhibits more robustness than the conventional models when the cohorts contain a certain amount of censored data. Here, we systematically evaluate the performance of our GuanRank-GPR model in a cross-cohort fashion: for each pair of cohorts, we trained our model on one cohort and validate the performance on the other. Figure 4 illustrates the evaluation of the results of 12 training-test pairs with integrated

AUC and C-index. All the values are over 0.5 and it shows that the model is robust to cohort bias.

Gene determinants of MM progression prediction are extracted from feature importance analysis

A key set of 342 gene determinants related to MM progression were identified based on the feature importance from random forests regression. To investigate the functional pathways associated with these genes, we performed functional enrichment analysis. After filtering with Q-value and fold enrichment, we found 69 significantly enriched GO biological processes (Supplementary Figure S1; Figure 5 shows the top 25 processes). Most processes are related with cell cycle or chromosomal instability. The cell cycle is a process by which cells progress, divide and reproduce themselves. Proper cell cycle progression is regulated by cell cycle proteins and checkpoint pathways. However, deregulation of cell cycle progression is one of the key hallmarks of cancer [32]. Chromosomal instability is another characteristic property of cancer cells, where chromosomes are not stable as they are in normal cells [33].

In order to better understand the molecular basis of the MM, we studied the gene interactions and the shared biological processes under a mammalian gene function network (Figure 6). We clustered the functionally related genes and different colors represent different clusters. Three clusters (cluster 3, 6, 7) are related with cell cycles, including cell division, nuclear division, regulation and transition of mitotic cell cycle. Other clusters are related with chromatin modification (cluster 2), spermatogenesis (cluster 1), ribosome biogenesis (cluster 4) and immune system (cluster 5).

Several genes in our gene signature were reported in the recent studies. *MYBL2* and *ANP32E* were identified as the top 2 important genes in our result. *MYBL2* is a gene which encodes a transcription factor with functions in checkpoint control of the G2 cell cycle phase. Heinrichs *et al.* considered this gene as a key tumor suppressor and believed it plays an important role in myeloid malignancies [34]. *ANP32E* is a member of the acid nuclear protein family that has been implicated in histone acetyltransferase inhibitory activity. Walker *et al.* identified ANP32E as one of prognostic important genes for myeloma in 372 MM patients [35]. Furthermore, several genes have never been reported to be associated with MM, but they are oncogenes (e.g. *TPX2* is related with gastric cancer [36,37] and pancreatic cancer [38]; *UBE2C* is related with prostate cancer [39]

and colorectal cancer [40]). Further validations for these genes are needed. The complete gene signature is available in the Supplementary Table S1.

Discussion

With the development of machine learning techniques, survival analysis can benefit more from the efforts of state-of-the-art algorithms. Many machine learning approaches as well as statistical models and their extensions were developed for survival prediction. In this paper, we used a non-parametric ranking method to assign a hazard score to each patient in the study. Then we built a Gaussian process regression model on the gene expression profile with the hazard scores as the target. Our model outperformed other popular models when evaluated using both integrated AUC and C-index of the predictions. The model also showed the robust predictive power in our cross-cohort validations.

There are three advantages of the GuanRank framework. First, it does not rely on any assumption, while Cox model assumes a proportional hazards condition [7]. Second, it is easy to generalize the data into a standard regression problem, where many machine learning methods can be applied to the survival prediction. Third, it completely ranks the patient pairs including ealy-censored-late-uncensored pairs, which is not considered in the Cox [7] and Random Survival Forests [11]. However, it also has several limitations. When an event happened in an unbalanced way within the cohort, e.g. 98% patients progressed during the observation period in the EMTAB4032 cohort, the performance of our model is not as good as expected. Another problem is that our model focuses on the hazard ranking. It would lose the specific time information and only use the event order between a pair of patients.

After combining clinical information, the performance was improved. This indicates that a gene expression profile alone is inadequate in predicting progression for MM, and it is helpful to add more progression related features. Although gene expression profile has been widely used for hazard risk prediction, it can not reflect the whole landscape of MM progression. We need to develop a more comprehensive predictive model with an integrated genomics approach. Cytogenetic abnormality is another important marker for MM progression prediction, and it has been reported to provide prognostic information [41]. The DREAM Challenges also provided the cytogenetic data, however, it contained many missing values, and the performance dropped when adding these cytogenetics

features. We believed that the model would have better predictive power when stacking more high-quality cytogenetic features.

Specifically, we wanted to extract the most informative genes from the gene expression profile in order to better predict the progression of newly diagnosed MM patients. We built a gene expression profile based prognostic signature with GuanRank hazard score as the target value. Part of the genes were reported to be associated with MM, and part of genes were not but correlated with other type of cancers. A few gene signatures were published in the recent years, such as EMC-92 [42], UAMS-70 [43], UAMS-80 [44], IFM-15 [45], MRC-IX [46] and HM-19 [47]. There is little overlap among these signatures, and they are also not included in our signature. It is shown that all the signatures have the cohort bias and cannot completely reflect the MM progression. More comprehensive studies are needed in the future.

Funding

This study was supported by research funding from: NSF CAREER: On-line Service for Predicting Protein Phosphorylation Dynamics Under Unseen Perturbations (NSF-US14-PAF07599); University of Michigan O'Brien Kidney Translational Core Center NIH (5-P30-DK-081943-10); Michigan Institute for Data Science (MIDAS) grant - Michigan Center for Single-Cell Genomic Data Analytics.

Acknowledgements

The 2017 Multiple Myeloma DREAM Challenge organizers and data contributors: Andrew Dervan (Celgene), Michael Mason (Sage Bionetworks), Fadi Towfic (Celgene), Gareth Morgan (UAMS), Brian Walker (UAMS), Dirk Hose (University of Heidelberg), Daniel Auclair (MMRF), Nikhil Munshi (DFCI), Mehmet Samur (DFCI), Kenneth Shain (Moffitt Cancer Center), Frank Schmitz (Celgene), Daniel Rozelle (Celgene), Konstantinos Mavrommatis (Celgene), Brian White (Sage Bionetworks), Thomas Yu (Sage Bionetworks), Justin Guinney (Sage Bionetworks), Doug Bassett (Celgene), Anjan Thakurta (Celgene).

Authorship Contributions

CS analyzed the data and drafted the manuscript. YG contributed the original winning solution. CS, HL, RM, YG edited the manuscript.

Disclosure of Conflicts of Interest

YG receives personal payment from Eli Lilly and Company, Genentech Inc, F. Hoffmann-La Roche AG, and Cleerly Inc; holds equity shares at Cleerly Inc and Ann Arbor Algorithms Inc; receives research support from Merck KGaA as research contracts and Ryss Tech as unrestricted donations.

Availability of supporting source code and requirements

Project name: Multiple Myeloma Survival Prediction

Project home page: https://www.synapse.org/#!Synapse:syn11459638

Includes: Data and dockerized environment for training and prediction

Operating system(s): Platform independent

Programming language: Perl, Python and Matlab

License: GNU GPL v3.0

An archival copy of code and data is also available via the GigaScience database GigaDB [48]. The dockerized environment has been registered in bio.tools with the identifier biotools: Multiple_Myeloma_survival_prediction, and in SciCrunch with the identifier RRID: SCR_017651.

References

1. Lionetti M, Neri A. Utilizing next-generation sequencing in the management of multiple myeloma. Expert Rev Mol Diagn. 2017;17:653–63.

2. Weaver CJ, Tariman JD. Multiple Myeloma Genomics: A Systematic Review. Semin Oncol Nurs. 2017;33:237–53.

3. Kyle RA, Rajkumar SV. Multiple myeloma. Blood. 2008;111:2962–72.

4. Siegel RL, Miller KD, Jemal A. Cancer statistics, 2018. CA Cancer J Clin. 2018;68:7–30.

5. Solly S. Remarks on the pathology of mollities ossium; with cases. Med Chir Trans. 1844;27:435–98.8.

6. Rajkumar SV. Multiple myeloma: 2016 update on diagnosis, risk-stratification, and management. Am J Hematol. 2016;91:719–34.

7. Cox DR. Regression Models and Life-Tables. Springer Series in Statistics. 1992. p. 527–41.

8. Tibshirani R. THE LASSO METHOD FOR VARIABLE SELECTION IN THE COX MODEL. Stat Med. 1997;16:385–95.

9. Verweij PJM, Van Houwelingen HC. Penalized likelihood in Cox regression. Stat Med. 1994;13:2427–36.

10. Simon N, Friedman J, Hastie T, Tibshirani R. Regularization Paths for Cox’s Proportional Hazards Model via Coordinate Descent. J Stat Softw [Internet]. 2011;39. Available from: http://dx.doi.org/10.18637/jss.v039.i05

11. Ishwaran H, Kogalur UB, Blackstone EH, Lauer MS. Random survival forests. Ann Appl Stat. 2008;2:841–60.

12. Stolovitzky G, Monroe D, Califano A. Dialogue on reverse-engineering assessment and methods: the DREAM of high-throughput pathway inference. Ann N Y Acad Sci. 2007;1115:1–22.

13. Huang Z, Zhang H, Boss J, Goutman SA, Mukherjee B, Dinov ID, et al. Complete hazard ranking to analyze right-censored data: An ALS survival study. PLoS Comput Biol. 2017;13:e1005887.

14. Mason M, Amatangelo M, Auclair D, Bassett D, Dai H, Dervan A, et al. Abstract 4725: Multiple Myeloma DREAM Challenge: A crowd-sourced challenge to improve identification of high-risk patients. Cancer Res. 2017;77:4725–4725.

15. Shi L, Campbell G, Jones WD, Campagne F, Wen Z, Walker SJ, et al. The MicroArray Quality Control (MAQC)-II study of common practices for the development and validation of microarray-based predictive models. Nat Biotechnol. 2010;28:827–38.

16. Broyl A, Hose D, Lokhorst H, de Knegt Y, Peeters J, Jauch A, et al. Gene expression

profiling for molecular classification of multiple myeloma in newly diagnosed patients. Blood. 2010;116:2543–53.

17. Kryukov F, Nemec P, Radova L, Kryukova E, Okubote S, Minarik J, et al. Centrosome associated genes pattern for risk sub-stratification in multiple myeloma. J Transl Med. 2016;14:150.

18. Multiple Myeloma Research Foundation. https://www.themmrf.org/. Accessed 12 March 2018 [Internet]. [cited 2018 Mar 12]. Available from: https://www.themmrf.org/

19. Greipp PR, San Miguel J, Durie BGM, Crowley JJ, Barlogie B, Bladé J, et al. International staging system for multiple myeloma. J Clin Oncol. 2005;23:3412–20.

20. GuanRank. http://guanlab.ccmb.med.umich.edu/yuanfang/Guan_rank.pdf. Accessed March 13, 2018 [Internet]. [cited 2018 Mar 13]. Available from: http://guanlab.ccmb.med.umich.edu/yuanfang/Guan_rank.pdf

21. Kaplan EL, Meier P. Nonparametric Estimation from Incomplete Observations. J Am Stat Assoc. 1958;53:457.

22. Rasmussen CE, Williams CKI. Gaussian Processes for Machine Learning. Mit Press; 2006.

23. Heagerty PJ, Zheng Y. Survival Model Predictive Accuracy and ROC Curves. Biometrics. 2005;61:92–105.

24. Harrell FE Jr, Lee KL, Mark DB. Multivariable prognostic models: issues in developing models, evaluating assumptions and adequacy, and measuring and reducing errors. Stat Med. 1996;15:361–87.

25. Blanche P, Dartigues J-F, Jacqmin-Gadda H. Estimating and comparing time-dependent areas under receiver operating characteristic curves for censored event times with competing risks. Stat Med. 2013;32:5381–97.

26. Pencina MJ, D'Agostino RB. OverallC as a measure of discrimination in survival analysis: model specific population value and confidence interval estimation [Internet]. Statistics in Medicine. 2004. p. 2109–23. Available from: http://dx.doi.org/10.1002/sim.1802

27. Amin SB, Yip W-K, Minvielle S, Broyl A, Li Y, Hanlon B, et al. Gene expression profile alone is inadequate in predicting complete response in multiple myeloma. Leukemia. 2014;28:2229–34.

28. Huang DW, Sherman BT, Lempicki RA. Systematic and integrative analysis of large gene lists using DAVID bioinformatics resources. Nat Protoc. 2009;4:44–57.

29. Guan Y, Myers CL, Lu R, Lemischka IR, Bult CJ, Troyanskaya OG. A Genomewide Functional Network for the Laboratory Mouse. PLoS Comput Biol. 2008;4:e1000165.

30. Newman MEJ, Girvan M. Finding and evaluating community structure in networks. Phys Rev E Stat Nonlin Soft Matter Phys. 2004;69:026113.

31. Zhu F, Panwar B, Dodge HH, Li H, Hampstead BM, Albin RL, et al. COMPASS: A computational model to predict changes in MMSE scores 24-months after initial assessment of Alzheimer's disease. Sci Rep [Internet]. 2016;6. Available from:

http://dx.doi.org/10.1038/srep34567

32. Maes A, Menu E, Veirman KD, Maes K, Vand Erkerken K, De Bruyne E. The therapeutic potential of cell cycle targeting in multiple myeloma. Oncotarget. 2017;8:90501–20.

33. Wang W, Zhang Y, Chen R, Tian Z, Zhai Y, Janz S, et al. Chromosomal instability and acquired drug resistance in multiple myeloma. Oncotarget. 2017;8:78234–44.

34. Heinrichs S, Conover LF, Bueso-Ramos CE, Kilpivaara O, Stevenson K, Neuberg D, et al. MYBL2 is a sub-haploinsufficient tumor suppressor gene in myeloid malignancy. Elife. 2013;2:e00825.

35. Walker BA, Leone PE, Chiecchio L, Dickens NJ, Jenner MW, Boyd KD, et al. A compendium of myeloma-associated chromosomal copy number abnormalities and their prognostic value. Blood. 2010;116:e56–65.

36. Tomii C, Inokuchi M, Takagi Y, Ishikawa T, Otsuki S, Uetake H, et al. TPX2 expression is associated with poor survival in gastric cancer. World J Surg Oncol. 2017;15:14.

37. Liang B, Zheng W, Fang L, Wu L, Zhou F, Yin X, et al. Overexpressed targeting protein for Xklp2 (TPX2) serves as a promising prognostic marker and therapeutic target for gastric cancer. Cancer Biol Ther. 2016;17:824–32.

38. Miwa T, Kokuryo T, Yokoyama Y, Yamaguchi J, Nagino M. Therapeutic potential of targeting protein for Xklp2 silencing for pancreatic cancer. Cancer Med. 2015;4:1091–100.

39. Lee CH, Ku JY, Ha JM, Bae SS, Lee JZ, Kim C-S, et al. Transcript Levels of Androgen Receptor Variant 7 and Ubiquitin-Conjugating Enzyme 2C in Hormone Sensitive Prostate Cancer and Castration-Resistant Prostate Cancer. Prostate. 2017;77:60–71.

40. Zhang Z, Liu P, Wang J, Gong T, Zhang F, Ma J, et al. Ubiquitin-conjugating enzyme E2C regulates apoptosis-dependent tumor progression of non-small cell lung cancer via ERK pathway. Med Oncol. 2015;32:149.

41. Kyle RA, Vincent Rajkumar S. Treatment of Multiple Myeloma: A Comprehensive Review. Clinical Lymphoma and Myeloma. 2009;9:278–88.

42. Kuiper R, Broyl A, de Knegt Y, van Vliet MH, van Beers EH, van der Holt B, et al. A gene expression signature for high-risk multiple myeloma. Leukemia. 2012;26:2406–13.

43. Shaughnessy JD Jr, Zhan F, Burington BE, Huang Y, Colla S, Hanamura I, et al. A validated gene expression model of high-risk multiple myeloma is defined by deregulated expression of genes mapping to chromosome 1. Blood. 2007;109:2276–84.

44. Shaughnessy JD Jr, Qu P, Usmani S, Heuck CJ, Zhang Q, Zhou Y, et al. Pharmacogenomics of bortezomib test-dosing identifies hyperexpression of proteasome genes, especially PSMD4, as novel high-risk feature in myeloma treated with Total Therapy 3. Blood. 2011;118:3512–24.

45. Decaux O, Lodé L, Magrangeas F, Charbonnel C, Gouraud W, Jézéquel P, et al. Prediction of survival in multiple myeloma based on gene expression profiles reveals cell cycle and chromosomal instability signatures in high-risk patients and hyperdiploid signatures in low-risk patients: a study of the Intergroupe Francophone du Myélome. J Clin Oncol. 2008;26:4798–805.

46. Dickens NJ, Walker BA, Leone PE, Johnson DC, Brito JL, Zeisig A, et al. Homozygous deletion mapping in myeloma samples identifies genes and an expression signature relevant to pathogenesis and outcome. Clin Cancer Res. 2010;16:1856–64.

47. Rème T, Hose D, Theillet C, Klein B. Modeling risk stratification in human cancer. Bioinformatics. 2013;29:1149–57.

48. Sun C, Li H, Mills RE, Guan Y: Supporting data for "Prognostic Model for Multiple Myeloma Progression Integrating Gene Expression and Clinical Features". GigaScience Database 2019. http://dx.doi.org/10.5524/100684

Tables

Table 1. Demographic characteristics of the four cohorts

Cohort	*GSE24080UAMS*	*HOVON65*	*MMRF*	*EMTAB4032*
**#Patient**	559	282	636	147
**Age (years)**	57.18±9.45	55.07±7.72	64.09±10.86	66.40±9.92
**Gender (M:F)**	1.52:1	1.33:1	1.46:1	1.10:1
**%Progression**	44.54%	66.67%	32.70%	97.99%
**Median Time to Progression (days)**	776.73	558.15	389.50	346.18
**%Death**	30.77%	34.75%	16.35%	36.91%
**Median Time to Death (days)**	830.62	532.23	402.50	1287.10
**%ISS I**	52.6%	40.1%	32.5%	25.5%
**%ISS II**	26.1%	25.2%	36.2%	30.2%
**%ISS III**	21.3%	28.7%	28.0%	40.3%

Figure Legends

Figure 1. **Overall workflow of the algorithm design to predict the progression of MM patients**. The original survival data were first converted into complete ranking scores via GuanRank. Four different cohorts were used to train models for predicting MM progression based on gene expression profiles and clinical features. The GPR method was employed in our final model to achieve the best prediction performance.

Figure 2. **Performance comparison of different survival analysis models in four cohorts.** In the GPR model, we directly use the progression status as the desired output to train, whereas in the GuanRank-GPR model we convert the progression status into a complete ranking score before training. The GuanRank-GPR model (red) consistently performed best in three cohorts (GSE24080UAMS, HOVON65 and MMRF) and second best in the EMTAB4032 cohort, when evaluated by both **A**) integrated AUC and **B**) C-index.

Figure 3. **Integrating gene expression profiles and clinical features improves the prediction performance**. Two types of models were built using either gene expression data or clinical data as features. After stacking the predictions from gene expression data and clinical data (red), the performance consistently increased in all cohorts when evaluated by **A**) integrated AUC and **B**) C-index, except for the integrated AUC of EMTAB4032 and the C-index of HOVON65.

Figure 4. **Cross-cohort evaluation of the GuanRank-GPR model.** To test the robustness of our model in a cross-cohort fashion, we train our GuanRank-GPR model on one cohort and make predictions on another cohort. Each row represents the training cohort and each column represents the test cohort. There are no data in the diagonal of the matrices, which represent the self-prediction. The predictions were evaluated using **A**) integrated AUC and **B**) C-index.

Figure 5. **GO enrichment analysis of important genes in predicting MM progression**. The top 25 significant GO biological processes were enriched from 342 key genes related with MM progression. In the left panel, different colors represent different classes of biological process. Most biological processes are related with cell cycle. In the right panel,

the dark bars are the negative logarithmic transformation of P-value, and the light bars are the counts of genes.

Figure 6. **Functional clusters in the MM progression gene network.** The gene clusters were shown in different colors and visualized using a mammalian gene-gene interaction network. The shared biological processes of selected clusters were labeled in rectangles.

Figure 1

Click here to access/download;Figure;figure1.png

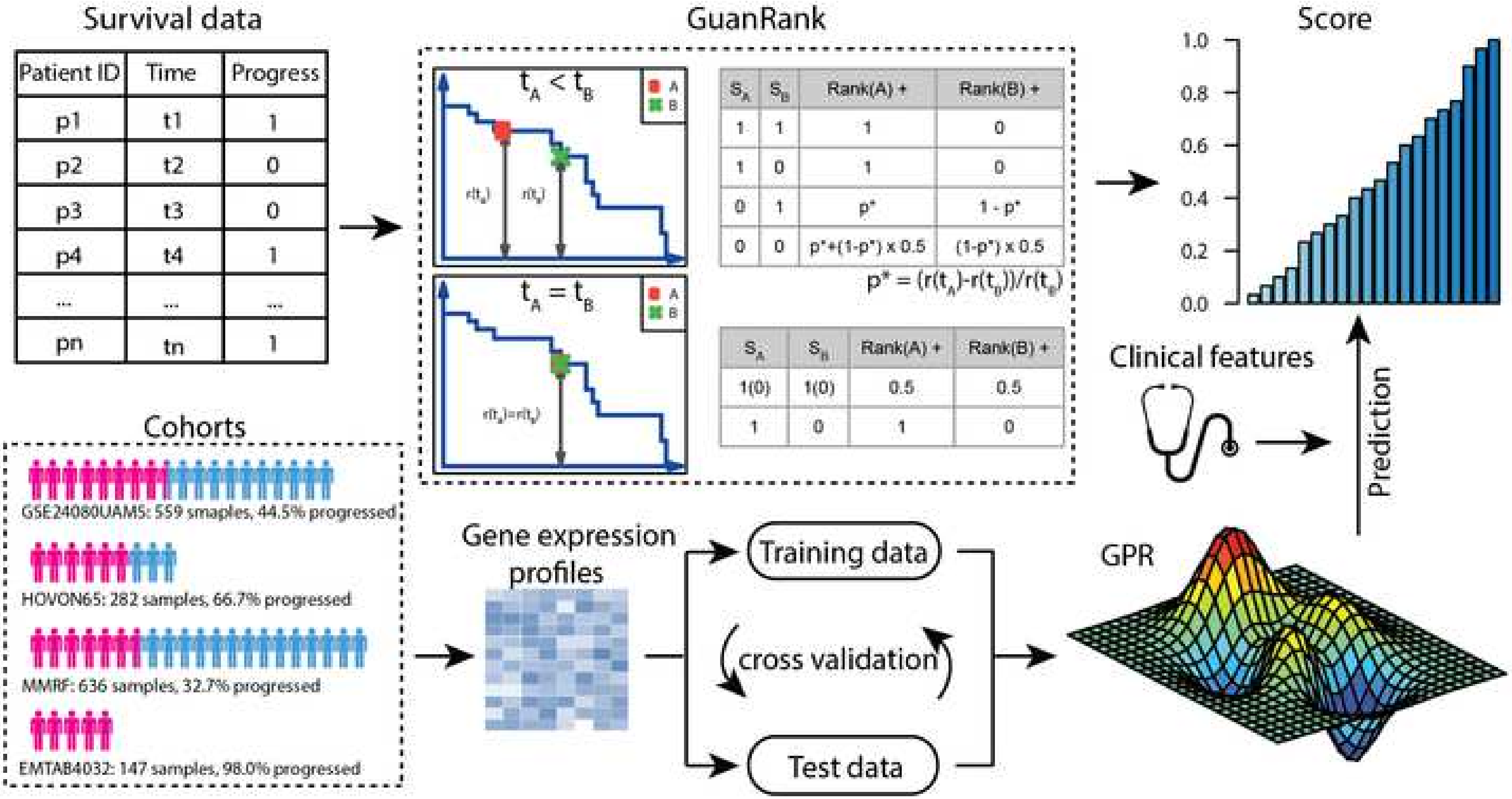

Figure 2

Click here to access/download;Figure;figure2.tif

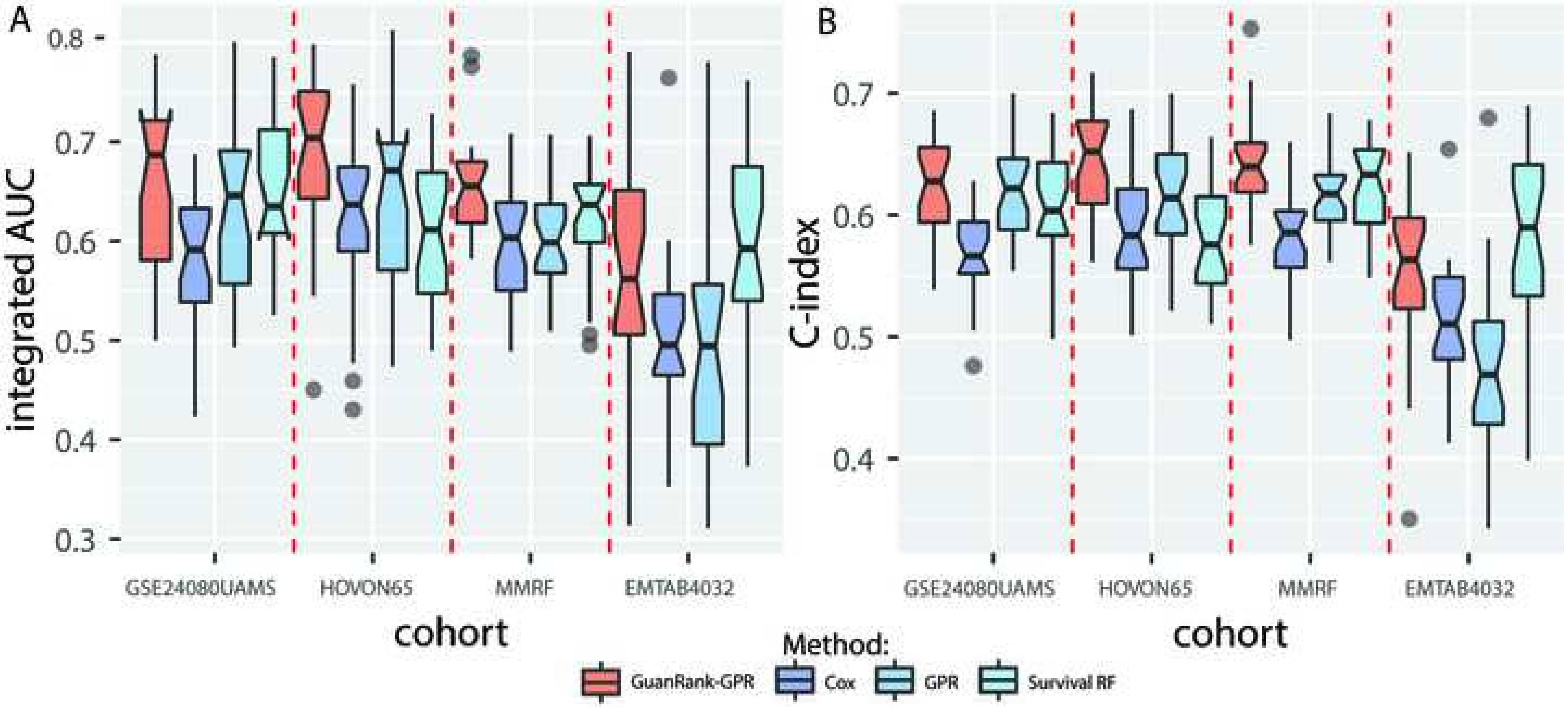

Figure 3

Click here to access/download;Figure;figure3.tif

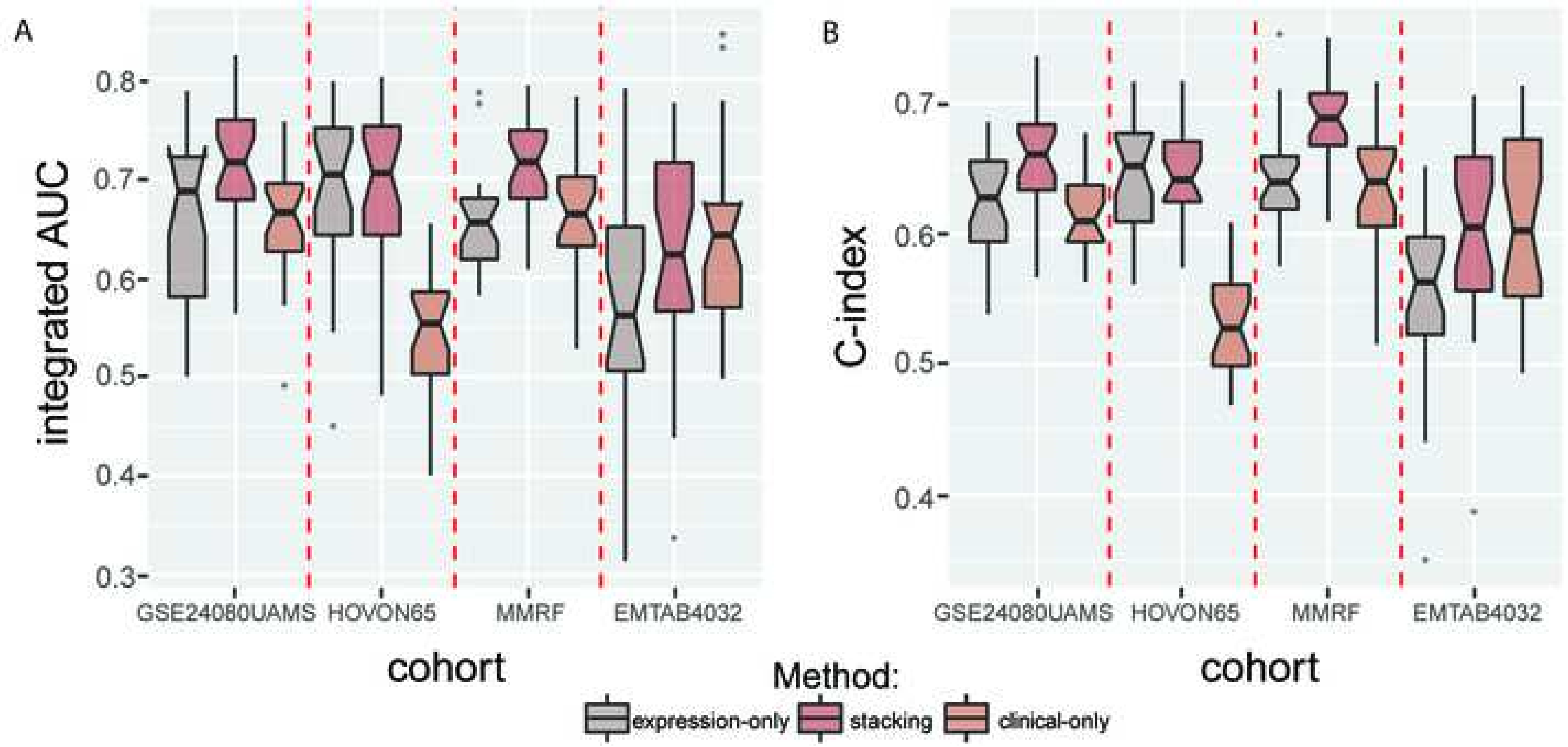

Figure 4

Click here to access/download;Figure;figure4.png

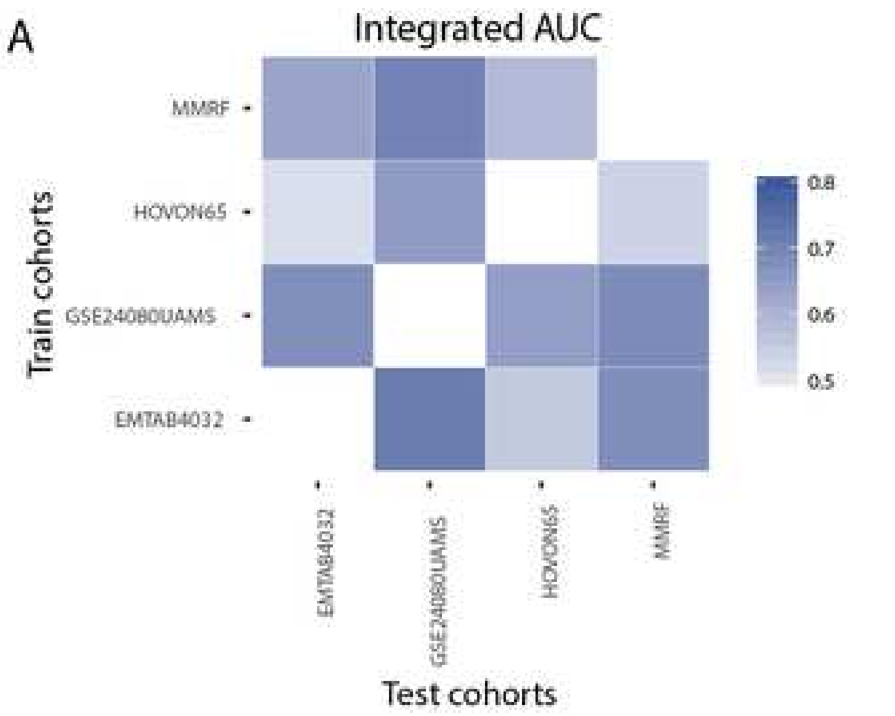

Figure 5

Click here to access/download;Figure;figure5.png

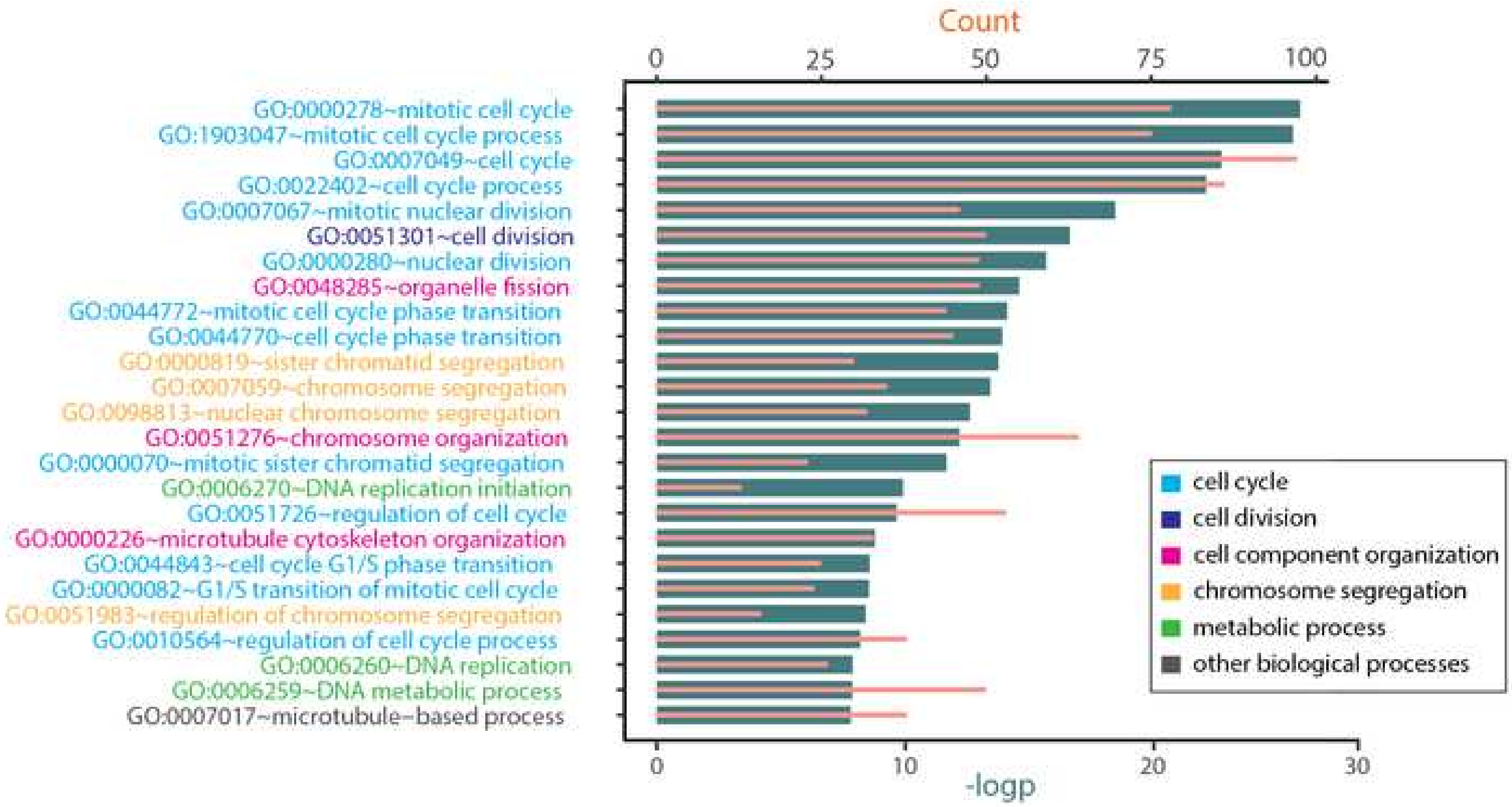

Figure 6

Click here to access/download;Figure;figure6.png

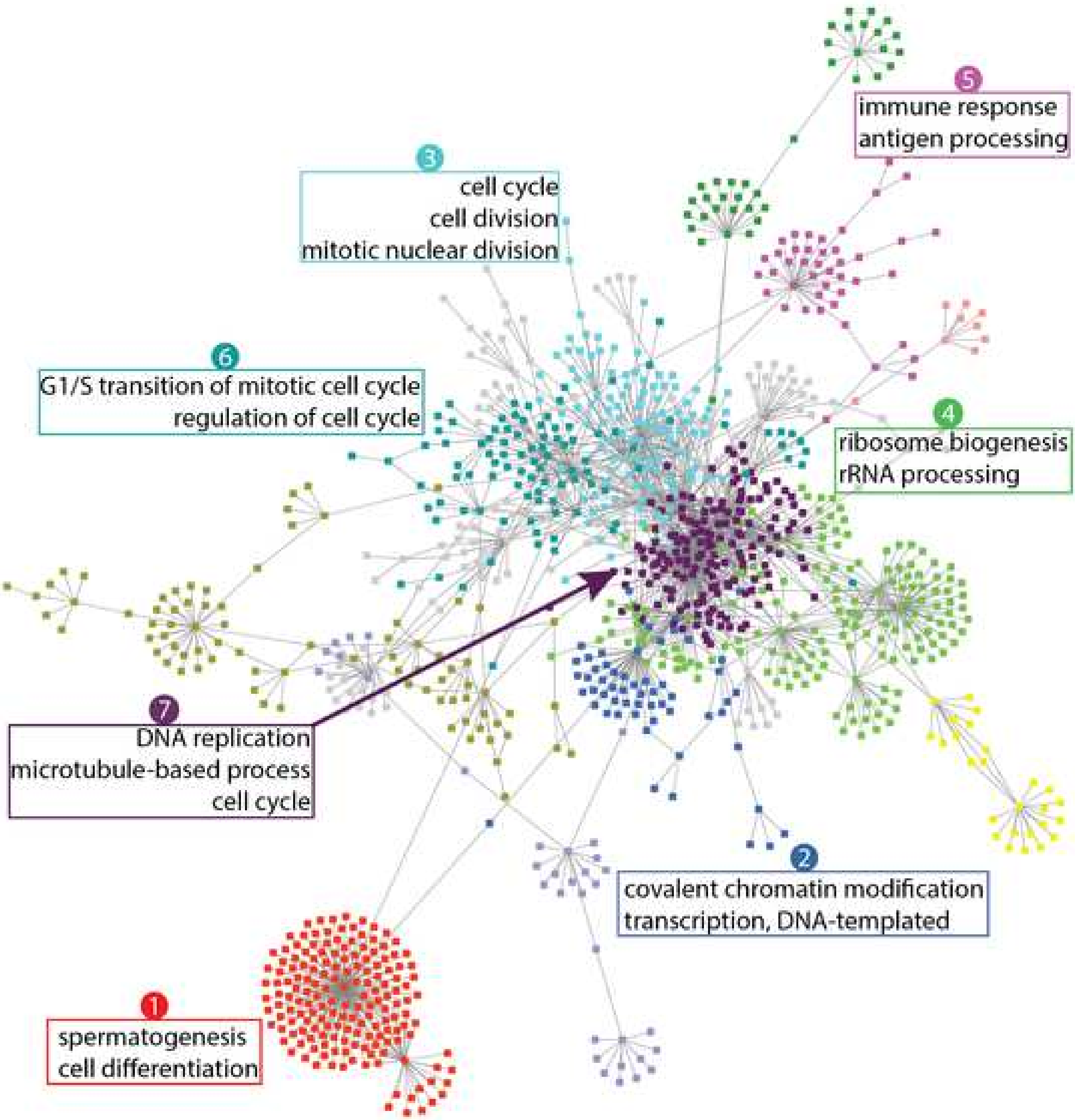

Click here to access/download
Supplementary Material
SupplementaryFiles.pdf

Response to the reviewer's comments

Click here to access/download
Supplementary Material
Response_to_reviewers_comments.pdf

Reviews of original submission

Click here to access/download

Supplementary Material

ReviewsOriginalSubmission.txt

Personal Cover

Click here to access/download;Personal Cover;cover_letter_MM.pdf

Dear editors,

We have submitted the revised version of our manuscript "Prognostic Model for Multiple Myeloma Progression Integrating Gene Expression and Clinical Features" (GIGA-D-18-00273) to the online system. We appreciate the thoughtful comments and suggestions of the reviewers, and are grateful for the submission guidance. A detailed point-by-point response to the comments of the reviewers is provided.

In addition, we want to mention that although the Multiple Myeloma DREAM challenge has three sub-challenges, we will not submit three separate papers - this manuscript is the only one. Before submitting this revised manuscript, we have been waiting for the challenge flagship paper online:
https://www.biorxiv.org/content/10.1101/737122v1

In conclusion, we thank you and the reviewers and hope our revised submission is suitable for publication in *GigaScience*.

Yours sincerely,

Yuanfang Guan
