# Supplementary material for: Prognostic model for multiple myeloma progression integrating gene expression and clinical features
Source: Gigascience. 2019 Dec 30;8(12):giz153. doi: 10.1093/gigascience/giz153 (PMC6936209; doi:10.1093/gigascience/giz153)
Supplement: giz153_Supplemental_Files [file giz153_supplemental_files.zip › SupplementaryFiles.pdf]

Supplementary Figure S1.

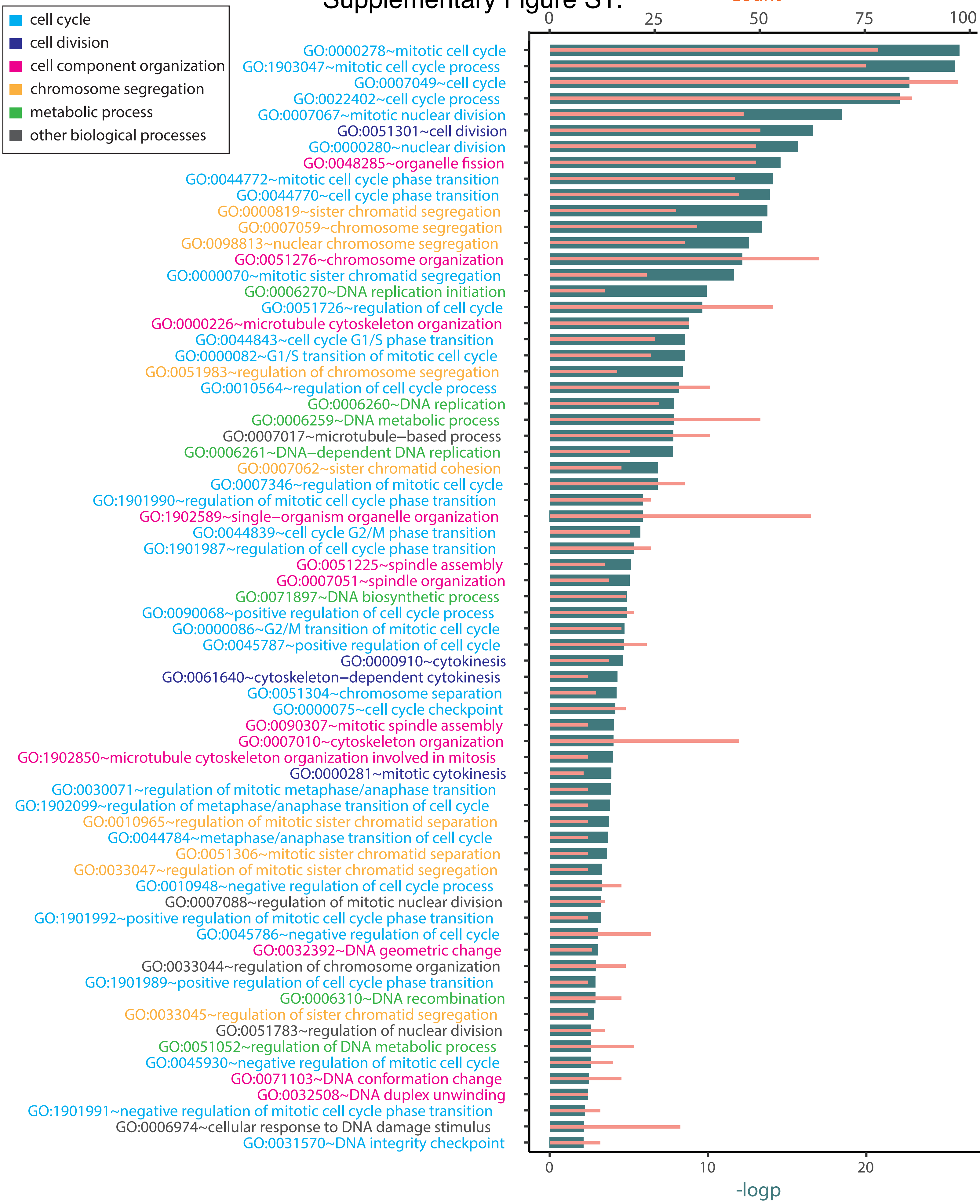

Supplementary Table S1. Complete gene list of MM progression

Entrez ID	Gene Symbo	Species	Gene Name
4605	MYBL2	Homo sapiens	MYB proto-oncogene like 2(MYBL2)
81611	ANP32E	Homo sapiens	acidic nuclear phosphoprotein 32 family member E(ANP32E)
79366	HMGN5	Homo sapiens	high mobility group nucleosome binding domain 5(HMGN5)
22974	TPX2	Homo sapiens	TPX2, microtubule nucleation factor(TPX2)
26147	PHF19	Homo sapiens	PHD finger protein 19(PHF19)
11065	UBE2C	Homo sapiens	ubiquitin conjugating enzyme E2 C(UBE2C)
55038	CDCA4	Homo sapiens	cell division cycle associated 4(CDCA4)
4174	MCM5	Homo sapiens	minichromosome maintenance complex component 5(MCM5)
23046	KIF21B	Homo sapiens	kinesin family member 21B(KIF21B)
2171	FABP5	Homo sapiens	fatty acid binding protein 5(FABP5)
4172	MCM3	Homo sapiens	minichromosome maintenance complex component 3(MCM3)
10802	sec24a	Homo sapiens	SEC24 homolog A, COPII coat complex component(SEC24A)
83540	NUF2	Homo sapiens	NUF2, NDC80 kinetochore complex component(NUF2)
220134	SKA1	Homo sapiens	spindle and kinetochore associated complex subunit 1(SKA1)
4171	MCM2	Homo sapiens	minichromosome maintenance complex component 2(MCM2)
55143	CDCA8	Homo sapiens	cell division cycle associated 8(CDCA8)
23094	SIPA1L3	Homo sapiens	signal induced proliferation associated 1 like 3(SIPA1L3)
3149	HMGB3	Homo sapiens	high mobility group box 3(HMGB3)
387103	CENPW	Homo sapiens	centromere protein W(CENPW)
4751	NEK2	Homo sapiens	NIMA related kinase 2(NEK2)
203068	TUBB	Homo sapiens	tubulin beta class I(TUBB)
9088	pkmyt1	Homo sapiens	protein kinase, membrane associated tyrosine/threonine 1(PKMYT1)
699	BUB1	Homo sapiens	BUB1 mitotic checkpoint serine/threonine kinase(BUB1)
9557	CHD1L	Homo sapiens	chromodomain helicase DNA binding protein 1 like(CHD1L)
10092	ARPC5	Homo sapiens	actin related protein 2/3 complex subunit 5(ARPC5)
23321	TRIM2	Homo sapiens	tripartite motif containing 2(TRIM2)
84668	FAM126A	Homo sapiens	family with sequence similarity 126 member A(FAM126A)
332	BIRC5	Homo sapiens	baculoviral IAP repeat containing 5(BIRC5)
4054	LTBP3	Homo sapiens	latent transforming growth factor beta binding protein 3(LTBP3)
63967	CLSPN	Homo sapiens	claspin(CLSPN)

8844	KSR1	Homo sapiens	kinase suppressor of ras 1(KSR1)
25963	TMEM87A	Homo sapiens	transmembrane protein 87A(TMEM87A)
1503	CTPS1	Homo sapiens	CTP synthase 1(CTPS1)
6259	RYK	Homo sapiens	receptor-like tyrosine kinase(RYK)
79915	ATAD5	Homo sapiens	ATPase family, AAA domain containing 5(ATAD5)
9928	KIF14	Homo sapiens	kinesin family member 14(KIF14)
84747	UNC119B	Homo sapiens	unc-119 lipid binding chaperone B(UNC119B)
27161	AGO2	Homo sapiens	argonaute 2, RISC catalytic component(AGO2)
146909	KIF18B	Homo sapiens	kinesin family member 18B(KIF18B)
257364	snx33	Homo sapiens	sorting nexin 33(SNX33)
139135	PASD1	Homo sapiens	PAS domain containing 1(PASD1)
7465	WEE1	Homo sapiens	WEE1 G2 checkpoint kinase(WEE1)
10112	KIF20A	Homo sapiens	kinesin family member 20A(KIF20A)
1617	DAZ1	Homo sapiens	deleted in azoospermia 1(DAZ1)
4173	MCM4	Homo sapiens	minichromosome maintenance complex component 4(MCM4)
8284	KDM5D	Homo sapiens	lysine demethylase 5D(KDM5D)
3964	LGALS8	Homo sapiens	galectin 8(LGALS8)
57835	slc4a5	Homo sapiens	solute carrier family 4 member 5(SLC4A5)
23456	abcb10	Homo sapiens	ATP binding cassette subfamily B member 10(ABCB10)
112483	SAT2	Homo sapiens	spermidine/spermine N1-acetyltransferase family member 2(SAT2)
5347	PLK1	Homo sapiens	polo like kinase 1(PLK1)
1870	E2F2	Homo sapiens	E2F transcription factor 2(E2F2)
57121	LPAR5	Homo sapiens	lysophosphatidic acid receptor 5(LPAR5)
686	BTD	Homo sapiens	biotinidase(BTD)
7083	TK1	Homo sapiens	thymidine kinase 1(TK1)
139886	SPIN4	Homo sapiens	spindlin family member 4(SPIN4)
3249	HPN	Homo sapiens	hepsin(HPN)
51053	GMNN	Homo sapiens	geminin, DNA replication inhibitor(GMNN)
157313	CDCA2	Homo sapiens	cell division cycle associated 2(CDCA2)
388662	SLC6A17	Homo sapiens	solute carrier family 6 member 17(SLC6A17)
27101	CACYBP	Homo sapiens	calcyclin binding protein(CACYBP)

55635	DEPDC1	Homo sapiens	DEP domain containing 1(DEPDC1)
29127	RACGAP1	Homo sapiens	Rac GTPase activating protein 1(RACGAP1)
1528	CYB5A	Homo sapiens	cytochrome b5 type A(CYB5A)
55165	CEP55	Homo sapiens	centrosomal protein 55(CEP55)
54657	UGT1A4	Homo sapiens	UDP glucuronosyltransferase family 1 member A4(UGT1A4)
80174	DBF4B	Homo sapiens	DBF4 zinc finger B(DBF4B)
388419	BTBD17	Homo sapiens	BTB domain containing 17(BTBD17)
29028	ATAD2	Homo sapiens	ATPase family, AAA domain containing 2(ATAD2)
10376	TUBA1B	Homo sapiens	tubulin alpha 1b(TUBA1B)
54892	NCAPG2	Homo sapiens	non-SMC condensin II complex subunit G2(NCAPG2)
5778	PTPN7	Homo sapiens	protein tyrosine phosphatase, non-receptor type 7(PTPN7)
8318	CDC45	Homo sapiens	cell division cycle 45(CDC45)
6703	SPRR2D	Homo sapiens	small proline rich protein 2D(SPRR2D)
8491	MAP4K3	Homo sapiens	mitogen-activated protein kinase kinase kinase kinase 3(MAP4K3)
65982	ZSCAN18	Homo sapiens	zinc finger and SCAN domain containing 18(ZSCAN18)
83461	CDCA3	Homo sapiens	cell division cycle associated 3(CDCA3)
27248	ERLEC1	Homo sapiens	endoplasmic reticulum lectin 1(ERLEC1)
11004	KIF2C	Homo sapiens	kinesin family member 2C(KIF2C)
2517	FUCA1	Homo sapiens	fucosidase, alpha-L- 1, tissue(FUCA1)
9816	URB2	Homo sapiens	URB2 ribosome biogenesis 2 homolog (S. cerevisiae)(URB2)
7905	REEP5	Homo sapiens	receptor accessory protein 5(REEP5)
646450	Arih2os	Homo sapiens	ariadne homolog 2 opposite strand(ARIH2OS)
10615	SPAG5	Homo sapiens	sperm associated antigen 5(SPAG5)
2034	EPAS1	Homo sapiens	endothelial PAS domain protein 1(EPAS1)
1995	ELAVL3	Homo sapiens	ELAV like RNA binding protein 3(ELAVL3)
1062	CENPE	Homo sapiens	centromere protein E(CENPE)
5872	RAB13	Homo sapiens	RAB13, member RAS oncogene family(RAB13)
3428	IFI16	Homo sapiens	interferon gamma inducible protein 16(IFI16)
9787	DLGAP5	Homo sapiens	DLG associated protein 5(DLGAP5)
50859	SPOCK3	Homo sapiens	SPARC/osteonectin, cwcv and kazal like domains proteoglycan 3(SPOCK3)
4841	NONO	Homo sapiens	non-POU domain containing, octamer-binding(NONO)

7264	TSTA3	Homo sapiens	tissue specific transplantation antigen P35B(TSTA3)
23768	FLRT2	Homo sapiens	fibronectin leucine rich transmembrane protein 2(FLRT2)
10045	SH2D3A	Homo sapiens	SH2 domain containing 3A(SH2D3A)
4176	MCM7	Homo sapiens	minichromosome maintenance complex component 7(MCM7)
9889	zbed4	Homo sapiens	zinc finger BED-type containing 4(ZBED4)
24137	KIF4A	Homo sapiens	kinesin family member 4A(KIF4A)
1869	E2F1	Homo sapiens	E2F transcription factor 1(E2F1)
9319	TRIP13	Homo sapiens	thyroid hormone receptor interactor 13(TRIP13)
10024	TROAP	Homo sapiens	trophinin associated protein(TROAP)
4878	NPPA	Homo sapiens	natriuretic peptide A(NPPA)
9898	UBAP2L	Homo sapiens	ubiquitin associated protein 2 like(UBAP2L)
11243	PMF1	Homo sapiens	polyamine modulated factor 1(PMF1)
6839	SUV39H1	Homo sapiens	suppressor of variegation 3-9 homolog 1(SUV39H1)
56203	LMOD3	Homo sapiens	leiomodin 3(LMOD3)
80117	ARL14	Homo sapiens	ADP ribosylation factor like GTPase 14(ARL14)
9452	ITM2A	Homo sapiens	integral membrane protein 2A(ITM2A)
1063	CENPF	Homo sapiens	centromere protein F(CENPF)
57469	PNMAL2	Homo sapiens	paraneoplastic Ma antigen family like 2(PNMAL2)
58515	SELENOK	Homo sapiens	selenoprotein K(SELENOK)
353355	ZNF233	Homo sapiens	zinc finger protein 233(ZNF233)
3838	KPNA2	Homo sapiens	karyopherin subunit alpha 2(KPNA2)
84296	GINS4	Homo sapiens	GINS complex subunit 4(GINS4)
3655	ITGA6	Homo sapiens	integrin subunit alpha 6(ITGA6)
4189	DNAJB9	Homo sapiens	DnaJ heat shock protein family (Hsp40) member B9(DNAJB9)
10733	PLK4	Homo sapiens	polo like kinase 4(PLK4)
3930	LBR	Homo sapiens	lamin B receptor(LBR)
9918	NCAPD2	Homo sapiens	non-SMC condensin I complex subunit D2(NCAPD2)
728833	FAM72D	Homo sapiens	family with sequence similarity 72 member D(FAM72D)
3070	HELLS	Homo sapiens	helicase, lymphoid-specific(HELLS)
55920	RCC2	Homo sapiens	regulator of chromosome condensation 2(RCC2)
56938	ARNTL2	Homo sapiens	aryl hydrocarbon receptor nuclear translocator like 2(ARNTL2)

10721	POLQ	Homo sapiens	DNA polymerase theta(POLQ)
64097	EPB41L4A	Homo sapiens	erythrocyte membrane protein band 4.1 like 4A(EPB41L4A)
10321	CRISP3	Homo sapiens	cysteine rich secretory protein 3(CRISP3)
89838	ULK4P1	Homo sapiens	ULK4 pseudogene 1(ULK4P1)
2286	FKBP2	Homo sapiens	FK506 binding protein 2(FKBP2)
29967	LRP12	Homo sapiens	LDL receptor related protein 12(LRP12)
63922	CHTF18	Homo sapiens	chromosome transmission fidelity factor 18(CHTF18)
972	CD74	Homo sapiens	CD74 molecule(CD74)
5589	PRKCSH	Homo sapiens	protein kinase C substrate 80K-H(PRKCSH)
55969	c20orf24	Homo sapiens	chromosome 20 open reading frame 24(C20orf24)
79071	ELOVL6	Homo sapiens	ELOVL fatty acid elongase 6(ELOVL6)
5270	SERPINE2	Homo sapiens	serpin family E member 2(SERPINE2)
2146	EZH2	Homo sapiens	enhancer of zeste 2 polycomb repressive complex 2 subunit(EZH2)
1026	CDKN1A	Homo sapiens	cyclin dependent kinase inhibitor 1A(CDKN1A)
2630	GBAP1	Homo sapiens	glucosylceramidase beta pseudogene 1(GBAP1)
27022	FOXD3	Homo sapiens	forkhead box D3(FOXD3)
7376	NR1H2	Homo sapiens	nuclear receptor subfamily 1 group H member 2(NR1H2)
9700	ESPL1	Homo sapiens	extra spindle pole bodies like 1, separase(ESPL1)
10877	CFHR4	Homo sapiens	complement factor H related 4(CFHR4)
91057	CCDC34	Homo sapiens	coiled-coil domain containing 34(CCDC34)
150967	PKI55	Homo sapiens	DKFZp434H1419(PKI55)
643616	MOP-1	Homo sapiens	MOP-1(MOP-1)
55916	NXT2	Homo sapiens	nuclear transport factor 2 like export factor 2(NXT2)
64321	SOX17	Homo sapiens	SRY-box 17(SOX17)
5728	PTEN	Homo sapiens	phosphatase and tensin homolog(PTEN)
6628	SNRPB	Homo sapiens	small nuclear ribonucleoprotein polypeptides B and B1(SNRPB)
22989	MYH15	Homo sapiens	myosin heavy chain 15(MYH15)
146956	EME1	Homo sapiens	essential meiotic structure-specific endonuclease 1(EME1)
644669	LOC644669	Homo sapiens	ankyrin repeat domain 30B pseudogene(LOC644669)
80745	THUMPD2	Homo sapiens	THUMP domain containing 2(THUMPD2)
140609	NEK7	Homo sapiens	NIMA related kinase 7(NEK7)

8407	TAGLN2	Homo sapiens	transgelin 2(TAGLN2)
79683	ZDHHC14	Homo sapiens	zinc finger DHHC-type containing 14(ZDHHC14)
27099	SND1-IT1	Homo sapiens	SND1 intronic transcript 1(SND1-IT1)
5426	POLE	Homo sapiens	DNA polymerase epsilon, catalytic subunit(POLE)
2023	ENO1	Homo sapiens	enolase 1(ENO1)
23194	FBXL7	Homo sapiens	F-box and leucine rich repeat protein 7(FBXL7)
1019	CDK4	Homo sapiens	cyclin dependent kinase 4(CDK4)
84898	plxdc2	Homo sapiens	plexin domain containing 2(PLXDC2)
1033	CDKN3	Homo sapiens	cyclin dependent kinase inhibitor 3(CDKN3)
5770	PTPN1	Homo sapiens	protein tyrosine phosphatase, non-receptor type 1(PTPN1)
9493	KIF23	Homo sapiens	kinesin family member 23(KIF23)
285830	HLA-F-AS1	Homo sapiens	HLA-F antisense RNA 1(HLA-F-AS1)
2036	EPB41L1	Homo sapiens	erythrocyte membrane protein band 4.1 like 1(EPB41L1)
64841	GNPNAT1	Homo sapiens	glucosamine-phosphate N-acetyltransferase 1(GNPNAT1)
23479	ISCU	Homo sapiens	iron-sulfur cluster assembly enzyme(ISCU)
2305	FOXM1	Homo sapiens	forkhead box M1(FOXM1)
4100	MAGEA1	Homo sapiens	MAGE family member A1(MAGEA1)
7504	XK	Homo sapiens	X-linked Kx blood group(XK)
284207	METRNL	Homo sapiens	meteorin like, glial cell differentiation regulator(METRNL)
643664	SLC35G6	Homo sapiens	solute carrier family 35 member G6(SLC35G6)
79075	DSCC1	Homo sapiens	DNA replication and sister chromatid cohesion 1(DSCC1)
84284	NTPCR	Homo sapiens	nucleoside-triphosphatase, cancer-related(NTPCR)
344558	SH3RF3	Homo sapiens	SH3 domain containing ring finger 3(SH3RF3)
23582	CCNDBP1	Homo sapiens	cyclin D1 binding protein 1(CCNDBP1)
7411	VBP1	Homo sapiens	VHL binding protein 1(VBP1)
1605	DAG1	Homo sapiens	dystroglycan 1(DAG1)
7468	whsc1	Homo sapiens	Wolf-Hirschhorn syndrome candidate 1(WHSC1)
220004	PPP1R32	Homo sapiens	protein phosphatase 1 regulatory subunit 32(PPP1R32)
9056	SLC7A7	Homo sapiens	solute carrier family 7 member 7(SLC7A7)
1690	COCH	Homo sapiens	cochlin(COCH)
4017	LOXL2	Homo sapiens	lysyl oxidase like 2(LOXL2)

3714	JAG2	Homo sapiens	jagged 2(JAG2)
84790	TUBA1C	Homo sapiens	tubulin alpha 1c(TUBA1C)
51514	DTL	Homo sapiens	denticleless E3 ubiquitin protein ligase homolog(DTL)
340152	ZC3H12D	Homo sapiens	zinc finger CCCH-type containing 12D(ZC3H12D)
55835	CENPJ	Homo sapiens	centromere protein J(CENPJ)
7298	TYMS	Homo sapiens	thymidylate synthetase(TYMS)
2297	FOXD1	Homo sapiens	forkhead box D1(FOXD1)
10772	SRSF10	Homo sapiens	serine and arginine rich splicing factor 10(SRSF10)
990	CDC6	Homo sapiens	cell division cycle 6(CDC6)
10312	TCIRG1	Homo sapiens	T-cell immune regulator 1, ATPase H+ transporting V0 subunit a3(TCIRG1)
7517	XRCC3	Homo sapiens	X-ray repair cross complementing 3(XRCC3)
56261	GPCPD1	Homo sapiens	glycerophosphocholine phosphodiesterase 1(GPCPD1)
64577	ALDH8A1	Homo sapiens	aldehyde dehydrogenase 8 family member A1(ALDH8A1)
1520	CTSS	Homo sapiens	cathepsin S(CTSS)
10970	CKAP4	Homo sapiens	cytoskeleton associated protein 4(CKAP4)
51154	MRTO4	Homo sapiens	MRT4 homolog, ribosome maturation factor(MRTO4)
54760	PCSK4	Homo sapiens	proprotein convertase subtilisin/kexin type 4(PCSK4)
4669	NAGLU	Homo sapiens	N-acetyl-alpha-glucosaminidase(NAGLU)
1460	CSNK2B	Homo sapiens	casein kinase 2 beta(CSNK2B)
1949	EFNB3	Homo sapiens	ephrin B3(EFNB3)
84513	PLPP5	Homo sapiens	phospholipid phosphatase 5(PLPP5)
4288	MKI67	Homo sapiens	marker of proliferation Ki-67(MKI67)
123099	DEGS2	Homo sapiens	delta 4-desaturase, sphingolipid 2(DEGS2)
51667	NUB1	Homo sapiens	negative regulator of ubiquitin like proteins 1(NUB1)
440279	UNC13C	Homo sapiens	unc-13 homolog C(UNC13C)
100033437	SNORD115-2	Homo sapiens	small nucleolar RNA, C/D box 115-2(SNORD115-2)
8852	AKAP4	Homo sapiens	A-kinase anchoring protein 4(AKAP4)
57181	SLC39A10	Homo sapiens	solute carrier family 39 member 10(SLC39A10)
105377138	LOC1053771	Homo sapiens	uncharacterized LOC105377138(LOC105377138)
5531	PPP4C	Homo sapiens	protein phosphatase 4 catalytic subunit(PPP4C)
261726	TIPRL	Homo sapiens	TOR signaling pathway regulator(TIPRL)

25823	TPSG1	Homo sapiens	tryptase gamma 1(TPSG1)
51696	HECA	Homo sapiens	hdc homolog, cell cycle regulator(HECA)
11285	B4GALT7	Homo sapiens	beta-1,4-galactosyltransferase 7(B4GALT7)
91687	CENPL	Homo sapiens	centromere protein L(CENPL)
5126	pcsk2	Homo sapiens	proprotein convertase subtilisin/kexin type 2(PCSK2)
80183	RUBCNL	Homo sapiens	RUN and cysteine rich domain containing beclin 1 interacting protein like(RUBCNL)
140875	LINC00028	Homo sapiens	long intergenic non-protein coding RNA 28(LINC00028)
3020	H3F3A	Homo sapiens	H3 histone family member 3A(H3F3A)
5937	RBMS1	Homo sapiens	RNA binding motif single stranded interacting protein 1(RBMS1)
1017	CDK2	Homo sapiens	cyclin dependent kinase 2(CDK2)
4175	MCM6	Homo sapiens	minichromosome maintenance complex component 6(MCM6)
29089	UBE2T	Homo sapiens	ubiquitin conjugating enzyme E2 T(UBE2T)
9375	TM9SF2	Homo sapiens	transmembrane 9 superfamily member 2(TM9SF2)
9156	EXO1	Homo sapiens	exonuclease 1(EXO1)
54832	VPS13C	Homo sapiens	vacuolar protein sorting 13 homolog C(VPS13C)
6700	SPRR2A	Homo sapiens	small proline rich protein 2A(SPRR2A)
84503	ZNF527	Homo sapiens	zinc finger protein 527(ZNF527)
284021	MILR1	Homo sapiens	mast cell immunoglobulin like receptor 1(MILR1)
127933	UHMK1	Homo sapiens	U2AF homology motif kinase 1(UHMK1)
3779	KCNMB1	Homo sapiens	potassium calcium-activated channel subfamily M regulatory beta subunit 1(KCNMB1)
147912	SIX5	Homo sapiens	SIX homeobox 5(SIX5)
9848	MFAP3L	Homo sapiens	microfibrillar associated protein 3 like(MFAP3L)
163589	TDRD5	Homo sapiens	tudor domain containing 5(TDRD5)
3925	STMN1	Homo sapiens	stathmin 1(STMN1)
10293	TRAIP	Homo sapiens	TRAF interacting protein(TRAIP)
64219	PJA1	Homo sapiens	praja ring finger ubiquitin ligase 1(PJA1)
9212	AURKB	Homo sapiens	aurora kinase B(AURKB)
27346	TMEM97	Homo sapiens	transmembrane protein 97(TMEM97)
56301	SLC7A10	Homo sapiens	solute carrier family 7 member 10(SLC7A10)
286343	LURAP1L	Homo sapiens	leucine rich adaptor protein 1 like(LURAP1L)
7421	VDR	Homo sapiens	vitamin D (1,25- dihydroxyvitamin D3) receptor(VDR)

8541	PPFIA3	Homo sapiens	PTPRF interacting protein alpha 3(PPFIA3)
100128191	TMPO-AS1	Homo sapiens	TMPO antisense RNA 1(TMPO-AS1)
1936	EEF1D	Homo sapiens	eukaryotic translation elongation factor 1 delta(EEF1D)
6894	TARBP1	Homo sapiens	TAR (HIV-1) RNA binding protein 1(TARBP1)
6653	SORL1	Homo sapiens	sortilin related receptor 1(SORL1)
10066	SCAMP2	Homo sapiens	secretory carrier membrane protein 2(SCAMP2)
81553	FAM49A	Homo sapiens	family with sequence similarity 49 member A(FAM49A)
89801	PPP1R3F	Homo sapiens	protein phosphatase 1 regulatory subunit 3F(PPP1R3F)
2280	FKBP1A	Homo sapiens	FK506 binding protein 1A(FKBP1A)
23742	NPAP1	Homo sapiens	nuclear pore associated protein 1(NPAP1)
204	AK2	Homo sapiens	adenylate kinase 2(AK2)
284751	LINC01270	Homo sapiens	long intergenic non-protein coding RNA 1270(LINC01270)
10042	HMGXB4	Homo sapiens	HMG-box containing 4(HMGXB4)
820	CAMP	Homo sapiens	cathelicidin antimicrobial peptide(CAMP)
4211	MEIS1	Homo sapiens	Meis homeobox 1(MEIS1)
84337	ELOF1	Homo sapiens	elongation factor 1 homolog(ELOF1)
100302273	MIR1254-1	Homo sapiens	microRNA 1254-1(MIR1254-1)
11009	IL24	Homo sapiens	interleukin 24(IL24)
713	C1QB	Homo sapiens	complement C1q B chain(C1QB)
80895	ILKAP	Homo sapiens	ILK associated serine/threonine phosphatase(ILKAP)
64130	LIN7B	Homo sapiens	lin-7 homolog B, crumbs cell polarity complex component(LIN7B)
11130	ZWINT	Homo sapiens	ZW10 interacting kinetochore protein(ZWINT)
5932	RBBP8	Homo sapiens	RB binding protein 8, endonuclease(RBBP8)
7112	TMPO	Homo sapiens	thymopoietin(TMPO)
259266	ASPM	Homo sapiens	abnormal spindle microtubule assembly(ASPM)
285178	LOC285178	Homo sapiens	uncharacterized LOC285178(LOC285178)
124602	KIF19	Homo sapiens	kinesin family member 19(KIF19)
6737	TRIM21	Homo sapiens	tripartite motif containing 21(TRIM21)
11221	DUSP10	Homo sapiens	dual specificity phosphatase 10(DUSP10)
3776	KCNK2	Homo sapiens	potassium two pore domain channel subfamily K member 2(KCNK2)
80178	c16orf59	Homo sapiens	chromosome 16 open reading frame 59(C16orf59)

22924	MAPRE3	Homo sapiens	microtubule associated protein RP/EB family member 3(MAPRE3)
55789	depdc1b	Homo sapiens	DEP domain containing 1B(DEPDC1B)
8907	AP1M1	Homo sapiens	adaptor related protein complex 1 mu 1 subunit(AP1M1)
285972	LINC00996	Homo sapiens	long intergenic non-protein coding RNA 996(LINC00996)
3608	ILF2	Homo sapiens	interleukin enhancer binding factor 2(ILF2)
58480	RHOU	Homo sapiens	ras homolog family member U(RHOU)
3015	H2AFZ	Homo sapiens	H2A histone family member Z(H2AFZ)
57446	NDRG3	Homo sapiens	NDRG family member 3(NDRG3)
5558	PRIM2	Homo sapiens	primase (DNA) subunit 2(PRIM2)
4435	CITED1	Homo sapiens	Cbp/p300 interacting transactivator with Glu/Asp rich carboxy-terminal domain 1(CITED1)
9750	FAM65B	Homo sapiens	family with sequence similarity 65 member B(FAM65B)
29128	UHRF1	Homo sapiens	ubiquitin like with PHD and ring finger domains 1(UHRF1)
10170	DHRS9	Homo sapiens	dehydrogenase/reductase 9(DHRS9)
51157	ZNF580	Homo sapiens	zinc finger protein 580(ZNF580)
51555	PEX5L	Homo sapiens	peroxisomal biogenesis factor 5 like(PEX5L)
55787	TXLNG	Homo sapiens	taxilin gamma(TXLNG)
78987	creld1	Homo sapiens	cysteine rich with EGF like domains 1(CRELD1)
2316	FLNA	Homo sapiens	filamin A(FLNA)
10253	SPRY2	Homo sapiens	sprouty RTK signaling antagonist 2(SPRY2)
767557	SYS1-DBNDD	Homo sapiens	SYS1-DBNDD2 readthrough (NMD candidate)(SYS1-DBNDD2)
11169	WDHD1	Homo sapiens	WD repeat and HMG-box DNA binding protein 1(WDHD1)
115811	IQCD	Homo sapiens	IQ motif containing D(IQCD)
9389	SLC22A14	Homo sapiens	solute carrier family 22 member 14(SLC22A14)
63935	PCIF1	Homo sapiens	PDX1 C-terminal inhibiting factor 1(PCIF1)
54973	CPSF3L	Homo sapiens	cleavage and polyadenylation specific factor 3-like(CPSF3L)
56180	MOSPD1	Homo sapiens	motile sperm domain containing 1(MOSPD1)
8505	PARG	Homo sapiens	poly(ADP-ribose) glycohydrolase(PARG)
26872	STEAP1	Homo sapiens	STEAP family member 1(STEAP1)
729991	BORCS8	Homo sapiens	BLOC-1 related complex subunit 8(BORCS8)
130162	CLHC1	Homo sapiens	clathrin heavy chain linker domain containing 1(CLHC1)
55893	ZNF395	Homo sapiens	zinc finger protein 395(ZNF395)

55258	THNSL2	Homo sapiens	threonine synthase like 2(THNSL2)
6804	stx1a	Homo sapiens	syntaxin 1A(STX1A)
5427	POLE2	Homo sapiens	DNA polymerase epsilon 2, accessory subunit(POLE2)
115353	LRRC42	Homo sapiens	leucine rich repeat containing 42(LRRC42)
5526	PPP2R5B	Homo sapiens	protein phosphatase 2 regulatory subunit B'beta(PPP2R5B)
5318	PKP2	Homo sapiens	plakophilin 2(PKP2)
9015	TAF1A	Homo sapiens	TATA-box binding protein associated factor, RNA polymerase I subunit A(TAF1A)
2271	FH	Homo sapiens	fumarate hydratase(FH)
79735	TBC1D17	Homo sapiens	TBC1 domain family member 17(TBC1D17)
54863	TOR4A	Homo sapiens	torsin family 4 member A(TOR4A)
55344	PLCXD1	Homo sapiens	phosphatidylinositol specific phospholipase C X domain containing 1(PLCXD1)
3679	itga7	Homo sapiens	integrin subunit alpha 7(ITGA7)
55768	NGLY1	Homo sapiens	N-glycanase 1(NGLY1)
10910	SUGT1	Homo sapiens	SGT1 homolog, MIS12 kinetochore complex assembly cochaperone(SUGT1)
54822	TRPM7	Homo sapiens	transient receptor potential cation channel subfamily M member 7(TRPM7)
221150	SKA3	Homo sapiens	spindle and kinetochore associated complex subunit 3(SKA3)
23076	RRP1B	Homo sapiens	ribosomal RNA processing 1B(RRP1B)
5985	RFC5	Homo sapiens	replication factor C subunit 5(RFC5)
3104	ZBTB48	Homo sapiens	zinc finger and BTB domain containing 48(ZBTB48)
79770	TXNDC15	Homo sapiens	thioredoxin domain containing 15(TXNDC15)
79086	SMIM7	Homo sapiens	small integral membrane protein 7(SMIM7)
100500849	MIR3916	Homo sapiens	microRNA 3916(MIR3916)
101928069	LOC1019280	Homo sapiens	uncharacterized LOC101928069(LOC101928069)
11099	PTPN21	Homo sapiens	protein tyrosine phosphatase, non-receptor type 21(PTPN21)
7514	XPO1	Homo sapiens	exportin 1(XPO1)
255101	CFAP65	Homo sapiens	cilia and flagella associated protein 65(CFAP65)
7068	THRB	Homo sapiens	thyroid hormone receptor beta(THRB)
11151	coro1a	Homo sapiens	coronin 1A(CORO1A)
7095	SEC62	Homo sapiens	SEC62 homolog, preprotein translocation factor(SEC62)
130355	c2orf76	Homo sapiens	chromosome 2 open reading frame 76(C2orf76)
4103	MAGEA4	Homo sapiens	MAGE family member A4(MAGEA4)

5202	PFDN2	Homo sapiens	prefoldin subunit 2(PFDN2)
2628	GATM	Homo sapiens	glycine amidinotransferase(GATM)
