# Supplementary material for: Prognostic model for multiple myeloma progression integrating gene expression and clinical features
Source: Gigascience. 2019 Dec 30;8(12):giz153. doi: 10.1093/gigascience/giz153 (PMC6936209; doi:10.1093/gigascience/giz153)
Supplement: giz153_Response_to_Reviewer_Comments_Original_Submission [file giz153_response_to_reviewer_comments_original_submission.pdf]

Author's Response To Reviewer Comments

Close

Dear editor,

Thank you for your comments. We have thoroughly revised the manuscript as the instructions.

Best wishes,
Yuanfang Guan

Close
