# Supplementary material for: Prognostic model for multiple myeloma progression integrating gene expression and clinical features
Source: Gigascience. 2019 Dec 30;8(12):giz153. doi: 10.1093/gigascience/giz153 (PMC6936209; doi:10.1093/gigascience/giz153)
Supplement: giz153_Supplemental_Files [file Response_to_reviewers_comments.pdf › Response_to_reviewers_comments.pdf]

Response to reviewers' comments
(Manuscript GIGA-D-18-00273)

We appreciate the reviewers for their insightful comments. The manuscript has been revised as the reviews advised. We have addressed all reviewers' comments thoroughly and provided a detailed point-by-point response as follows.

Comments from Reviewer #1

While Stacking, as expected, outperforms expression only, I am having a problem understanding the benefit of GR-GPR versus Survival Random Forest; it seems to generally do no better. Can you expand on this? Is there a way to combine the two to get a method that always outperforms the individual methods? Can you argue why one does better than the other or differently than the other? If this is possible, I would fully support the publication of this manuscript.

A: We thank the reviewer's support towards the publication of this article, and have carefully addressed the remaining concerns below.

In Figure 2, we compared the prediction performance of the GuanRank-GPR model and Survival Random Forest in 4 cohorts. The GuanRank-GPR model performed best in the three (GSE24080UAMS, HOVON65 and MMRF) out of four cohorts. In the EMTAB4032 cohort, our model performed slightly worse than survival random forests. In fact, most patients (143, 98%) in this cohort progressed during the longitudinal observation period, while the progression rates in the other cohort are only 44.5%, 66.7% and 32.7%. The GuanRank approach was designed to integrate the censoring information of patients without progression and calculate the rank score. When the percentage of right-censored patients was extremely small such as 2% in the EMTAB4032 cohort, the advantage of GuanRank was lost. But in general, the GuanRank-GPR model has demonstrated better performance than survival random forests.

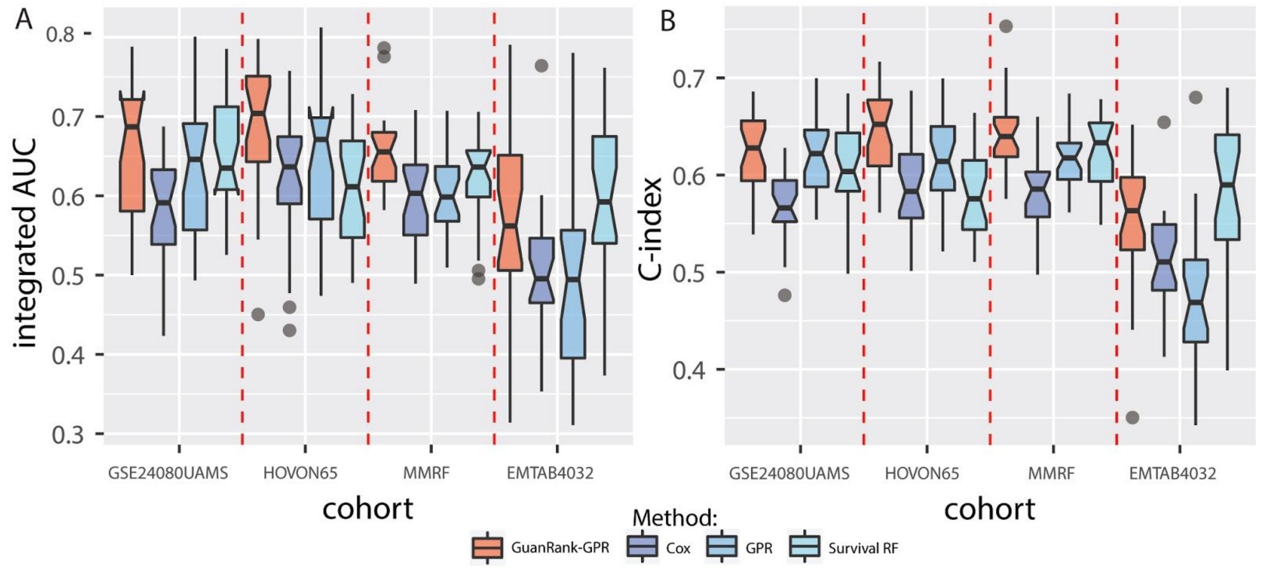

Figure 2. Performance comparison of different survival analysis models in four cohorts. In the GPR model, we directly use the progression status as the desired output to train, whereas in the GuanRank-GPR model we convert the progression status into a complete ranking score before training. The GuanRank-GPR model (red) consistently performed best in three cohorts (GSE24080UAMS, HOVON65 and MMRF) and second best in the EMTAB4032 cohort, when evaluated by both A) integrated AUC and B) C-index.

Random survival forests calculated the log-rank of patients within a short time interval (figure below). However, when patients were censored within the selected interval, they were treated equally as patients censored later. In fact, a late-censored patient at time “T-late” was expected to have a lower risk than a early-censored patient at time “T-early”, since the early-censored patient could potentially had the disease progression any time between “T-early” and “T-late”. In contrast to random survival forests, GuanRank had a pairwise comparison of all patients, fully comparing all the censoring events. Therefore, our method generally had an advantage over random survival forests.

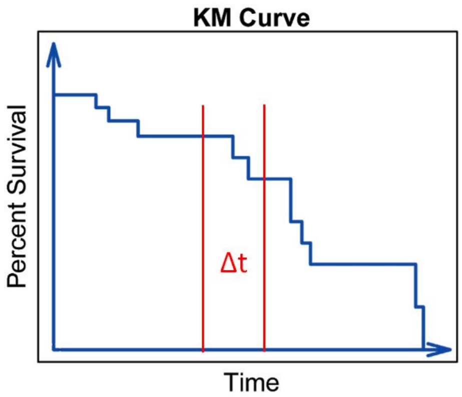

Building an ensemble model to combine the two methods is an excellent idea.
add figure (e.g. 0.8 vs 0.2 weights)

Comments from Reviewer #2

This manuscript presents the application of an existing non-parametric ranking method (GuanRank) to build prognostic models for Multiple Myeloma (MM) from gene expression and clinical features. This is reporting the authors's work on the DREAM challenge MM Challenge.
I have struggled to understand which contributions are novel. In part, this is due to many parts not being well described and/or motivated:

* Page 3: the reason why early-censored condition is considered in COX models or the definition censored data should be here, not later in the manuscript.

A: Thanks for pointing this out. We agree with the reviewer that the definition of censored data should be mentioned earlier. We therefore added the definition of the censoring problem and right-censored data in the second paragraph of the background section as follows:

"A major challenge of analyzing time-to-event data is the censoring problem - the patient status is not fully available due to tracking interruption or time limit of a study. In this work, we focused on the right-censored data, in which the censored patient did not have disease progression at the censoring time but his/her future status was not available."

We also described the reason that early-censored condition was not considered in Cox models in the same paragraph as follows:

"However, Cox model only optimize the partial maximum likelihood function of all realized events without considering the likelihood of censored patients. In addition, Cox models require multiple assumptions that may not be met in many real situations."

* Page 3: the authors claim that this is the best-performing method by predictive accuracy in the challenge, but results show that they were only the top performers in subchallenge 3. As usual, the difference between top performers is quite small. Not the case in subchallenges 1 or 2:

https://www.synapse.org/#!Synapse:syn6187098/wiki/499425 This must be fully explained in the manuscript.

A: Thanks for pointing this out. We have now added "Sub-Challenge 3" in the manuscript to clarify this. In fact, among the three sub-challenges, Sub-Challenge 3 is the most comprehensive sub-challenge and the features of Sub-Challenge 1 and 2 are included in Sub-Challenge 3. From the DREAM website, the designs of three sub-challenges are:
Sub-Challenge 1: "prediction of clinical progression by 18 months using DNA-based features only (with the option to include ISS and age)";
Sub-Challenge 2: "prediction of clinical progression by 18 months using RNA-based features only (with the option to include ISS and age)" ;
Sub-Challenge 3: "prediction of clinical progression by 18 months using any combination of DNA-based features, RNA-based features, age, ISS, and other demographic and clinical or cytogenic variables".

* Incidentally, the study must report all three subchallenges. There is no justification to hack this study into three separate papers when they refer to exactly the same data.

A: We thank the reviewer's comments. In fact, we only want to publish our top-performing algorithm for Sub-Challenge 3 (instead of three separate papers). There is a flagship paper about this DREAM challenge covering three sub-challenges. This paper is available at bioRxiv: https://www.biorxiv.org/content/10.1101/737122v1.

* Page 4: Are they using the data from different sources directly or after being pre-processed by DREAM? How did they ensure consistency between the four gene expression data sets?

A: All the gene expression data were pre-processed by the DREAM challenge organizers and data providers. We further imputed the missing values using the mean value across patients and quantile normalized the expression data to ensure consistency and eliminate potential batch effects.

* Page 4: quantile-normalized the expression data: how? Reference? Per-gene? Per-patient?

A: Per patient. For each patient, we first ranked the expression levels of all genes and obtained the ranked gene expression profile of a patient. Then we calculated the average of the ranked profiles from all patients and used this average as the reference profile. Finally, we quantile normalized the gene expression profile of each patient based on the reference profile. Therefore, the gene expression profile followed the same distribution as the reference profile after the

quantile normalization.

*   Page 4: uniform in which way?

A: We apologize for the misleading usage of "uniform". We have now re-written this section on Page 5 as follows:

"To leverage cutting edge machine learning techniques, we need to transform label of the time-to-event data to a new regressible label. In particular, the original label of a patient contains two values: (i) the binary status and (ii) the corresponding event/censoring time. This leads to the common problem that the event/censoring time can not be used directly by typical machine learning models. Therefore, the desired new label needs to be a single value integrating the information of both the status and the time."

*   Page 5: writing is confusing here. Please use patient always (not sample, not data point). Rank of A seems a score using to rank patients, not the rank of the patient.

A: Thank you for this suggestion.. We have now used "patient" throughout the manuscript. We have changed the "rank of A" to "rank score of A".

*   Page 5: The idea behind GuanRank is essentially to use the normalized ranks of the patient as the dependent variable to predict, which poses it as a regression problem. It is surprising that no one has thought before of doing something so simple (yet effective). The authors seem to be the first, which is commendable. However, the method is not introduced here, they have already published a number of papers with it: I am missing all these references.

A: Thank you for your appreciation of the GuanRank idea. We have only one publication related to GuanRank and it is cited as the reference 13 on Page 4:

"13. Huang Z, Zhang H, Boss J, Goutman SA, Mukherjee B, Dinov ID, et al. Complete hazard ranking to analyze right-censored data: An ALS survival study. PLoS Comput Biol. 2017;13:e1005887."

*   Page 6: AUC is standard, how time-dependence concordance is calculated or the role of the weights is not explained at all. Same goes for C-index. The methods section must show the expression of these metrics, which are central to the evaluation. How you go from a continuous prediction to the binary prediction that AUC requires?

A: We are sorry about not explaining this clearly. We agree with the reviewer that detailed definition of time-dependent integrated AUC and C-index should be provided to readers. We have therefore added these details in the Method section on Page 6 as follows:

"Integrated AUC measures time-dependent concordance with the weights derived from the survival time distribution. For continuous predictions, a cutoff can be used to binarize the predictions and calculate one point in the ROC curve. The cutoff for continuous predictions gradually increases from 0 to 1 to obtain the ROC curve and corresponding AUC. The time-dependent AUC was first calculated at 14, 16, 18, 20, and 22 months using the weights from Kaplan-Meier estimator of the censoring distribution[25]. Then the integrated AUC was calculated from time-dependent AUC using the weights from 2*S(t)*f(t), where S(t) denoted the survival function and f(t) denoted the marginal density of the survival time Ti as described by Heagerty et al.[23] C-index estimates the probability that a randomly selected patient who has experienced an event has a higher risk score than a patient who has not experienced the event[26]. Both metrics are the measures of the goodness-of-fit for the survival model."

.

* Page 6: it is not stated how many gene expression features are there per cohort and how many of these are used as features. Same goes for clinical features.

A: Thanks for pointing this out. We have now provided this information under "Data collection" section on Page 4 as follows:

"There are 18,994, 20,514, 20,514 and 24128 gene expression features for the cohort EMTAB4032, GSE24080UAMS, HOVON65 and MMRF, respectively. All of the genes are used as features."

All cohorts only have two clinical features, age and ISS. We also mentioned this under the same section.

* Page 6: risk genes, risk of what exactly? Random forest is used now, instead of GPRs: why? The importance threshold of 0.01 is seems arbitrary, please motivate.

A: Thank you for the insightful questions. Risk genes are genes that are highly correlated with the progression of MM. GPR model was well suited for this problem because it modeled the similarity of different individuals in multiple cohorts. However, GPR was not ideal to evaluate the feature importance. On the other hand, although random forest was not perfect for this problem, it could naturally provide feature importance. We also tested the threshold 0.02 (146 genes), 0.05 (47 genes) and 0.001 (5175 genes). The sizes of these gene lists are not ideal for the GO enrichment analysis. We also tested other thresholds, but the number of genes is either too

small (47 genes; threshold=0.05) or too large (5175 genes; threshold=0.001). We hope that we have clarified these questions and the reviewer would agree with us on this point.

* Page 6: a major issue with gene lists is their lack of robustness with respect to different data sets. Using the same threshold, how many of the 342 genes are among the most important in all four cohorts? This should be discussed.

A: Thanks for pointing this out. The 342 genes are selected from the weighted summation of the four cohorts. We have provided the detailed calculation method on Page 7 as follows:

"Using the proportions of each cohort among the four cohorts (number of patients in one cohort / total number of patients in four cohorts) as the weights, we calculated the combined importance value for each gene. After sorting, we selected 342 genes whose importance values are higher than 0.01."

* Page 8: define progression rate.

A: Progression rate is the number of progressed patient over the number of total patients. We have now added this definition on Page 9.

* Page 9: how the presented validation is different from that requested by the DREAM challenge?

A: We used data from four different cohorts (A,B,C, and D) in this study. The DREAM challenge used the data from the fifth cohort (E) as the final test dataset, which was not publicly available. We performed cross-cohort validation using the available four cohorts and evaluated the performance using 5 times 5-fold cross-validation experiments. This cross-cohort design is similar the the DREAM challenge, reflecting the predictive performance of our model in a new cohort.

* Page 9: training on a feature vector including both gene expression and clinical features, how performance compares to expression-only, clinical-only and stacking models?

A: Thank you for this instructive suggestion. We agree with the reviewer and have therefore added the performance of clinical-only model on Figure 3 below. After stacking the predictions from gene expression data and clinical data (red), the performance consistently increased in all cohorts when evaluated by A) integrated AUC and B) C-index, except for the integrated AUC of EMTAB4032 and the C-index of HOVON65.

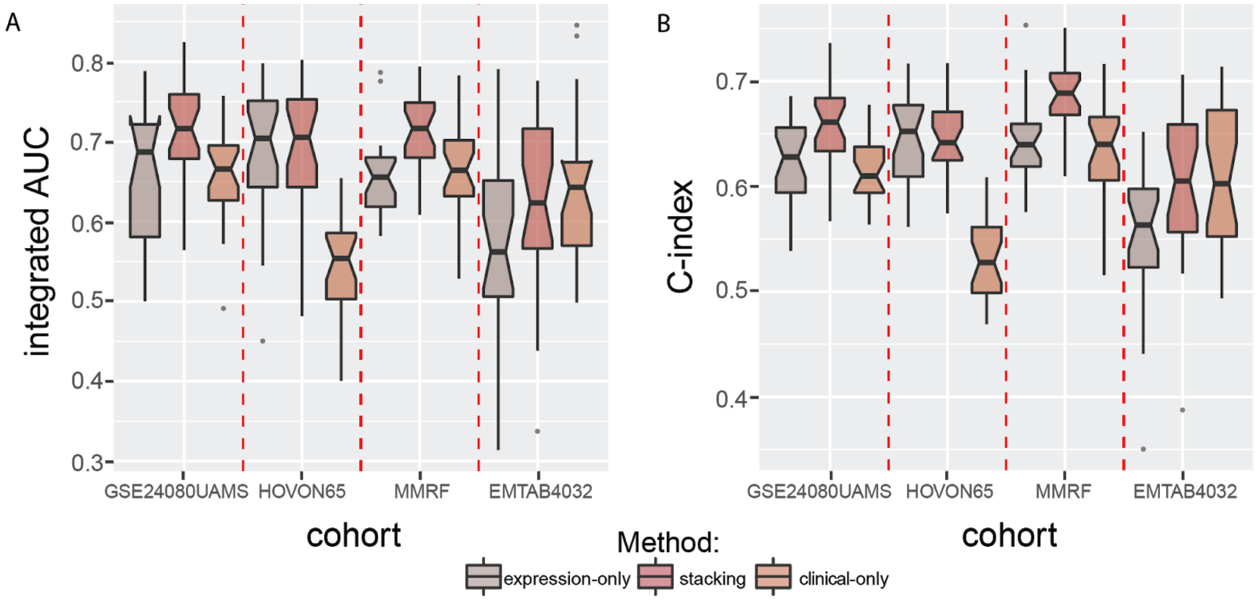

*   Page 9: what do you mean exactly by cohort and batch effects in this context?

A: A cohort is a group of people who may share a common characteristic or experience within a defined period. Different cohorts may have unique characteristics. Batch effects are due to technical differences between different sample sets, and they reflect the variability that is not our interest.

*   Page 9: 12 pairs should be 12 training-test pairs.

A: We agree and have modified it.

*   Page 10: why not human instead of mammalian gene function network?

A: Thanks for pointing this out. Phenotype annotations were mainly identified in mammalian model organisms and then computationally transferred to human annotation. The network we used also contained phenotype annotations in human from OMIM database. Therefore, networks based on human information only may not be as comprehensive as the mammalian network used in this study.

*   Page 11: Why GO enrichment is used? The authors should carry out GSEA to find out which cancer pathways are driving survival.

A: We agree with the reviewer that GSEA analysis should also be carried out to find enriched functional pathways. We therefore performed the GSEA analysis on three cohorts (the experiment information of MMRF cohort was not provided by the challenge organizers). The enriched gene sets were ranked by the normalized enrichment score (NES):

$$NES = \frac{actual\ enrichment\ score}{mean(enrichment\ scores\ against\ all\ permutations\ of\ the\ dataset)}$$

The detailed information about NES can be found at:
http://www.gsea-msigdb.org/gsea/doc/GSEAUserGuideFrame.html?_Interpreting_GSEA_Results

The top 20 enriched gene sets of the three cohorts are listed below. Many previous studies revealed that multiple myeloma is a disease of an immune system cell. It suppresses the immune response and therefore reducing the number of normal antibodies and affecting all the cells that would attack abnormal cells.[1-3] Here we label the immune system related gene sets as red.

Reference:

1. Kumar, Shaji K., and Kenneth C. Anderson. "Immune therapies in multiple myeloma." (2016): 5453-5460.
2. Pratt, Guy, Oliver Goodyear, and Paul Moss. "Immunodeficiency and immunotherapy in multiple myeloma." British journal of haematology 138.5 (2007): 563-579.
3. Giannopoulos, K., et al. "The frequency of T regulatory cells modulates the survival of multiple myeloma patients: detailed characterisation of immune status in multiple myeloma." British Journal of Cancer 106.3 (2012): 546.

cohort EMTAB:

GSE17974_IL4_AND_ANTI_IL12_VS_UNTREATED_24H_ACT_CD4_TCELL_DN
GO_POSITIVE_REGULATION_OF_CYTOKINE_SECRETION
GO_REGULATION_OF_MYOBLAST_DIFFERENTIATION
GSE23321_CENTRAL_MEMORY_VS_NAIVE_CD8_TCELL_DN
GO_REGULATION_OF_SKELETAL_MUSCLE_FIBER_DEVELOPMENT
GO_POSITIVE_REGULATION_OF_MYOTUBE_DIFFERENTIATION
GO_PROTEIN_TRANSPORT_WITHIN_LIPID_BILAYER
GSE19198_CTRL_VS_IL21_TREATED_TCELL_1H_UP
GO_CELL_CELL_ADHESION_INVOLVED_IN_GASTRULATION
GO_PRESYNAPTIC_MEMBRANE
GO_SOMITOGENESIS
GO_SOMITE_DEVELOPMENT
GO_NEGATIVE_REGULATION_OF_BLOOD_PRESSURE
GSE3039_ALPHAALPHA_CD8_TCELL_VS_B1_BCELL_DN
GO_IMMUNOGLOBULIN_PRODUCTION
GO_CELL_CHEMOTAXIS
GO_APPENDAGE_MORPHOGENESIS
GO_POSITIVE_REGULATION_OF_RESPONSE_TO_EXTERNAL_STIMULUS
GO_INTERLEUKIN_10_PRODUCTION
GO_REGULATION_OF_POSTSYNAPTIC_MEMBRANE_NEUROTRANSMITTER_RECEPTOR_LEVELS

cohort GSE24080UAMS:

GSE24634_IL4_VS_CTRL_TREATED_NAIVE_CD4_TCELL_DAY5_UP

GO_CELL_CELL_ADHESION
GSE40274_CTRL_VS_FOXP3_AND_LEF1_TRANSDUCED_ACTIVATED_CD4_TCELL_UP
ALK_DN.V1_DN
GSE3720_LPS_VS_PMA_STIM_VD2_GAMMADELTA_TCELL_DN
GSE7509_UNSTIM_VS_TNFA_IL1B_IL6_PGE_STIM_DC_DN
GSE37301_CD4_TCELL_VS_GRANULOCYTE_MONOCYTE_PROGENITOR_DN
GSE3203_HEALTHY_VS_INFLUENZA_INFECTED_LN_BCELL_DN
GSE27786_NKCELL_VS_ERYTHROBLAST_DN
GSE41176_UNSTIM_VS_ANTI_IGM_STIM_BCELL_3H_UP
GSE20366_TREG_VS_TCONV_UP
GSE22601_DOUBLE_POSITIVE_VS_CD4_SINGLE_POSITIVE_THYMOCYTE_UP
GO_SYNAPTIC_VESICLE_CYCLE
GO_REGULATION_OF_SYNAPTIC_PLASTICITY
CHR8P11
BRN2_01
GO_REGULATION_OF_CELL_ACTIVATION
ATF2_UP.V1_DN
HALLMARK_BILE_ACID_METABOLISM
GSE37336_LY6C_POS_VS_NEG_NAIVE_CD4_TCELL_UP

cohort HOVON65:
GSE21546_UNSTIM_VS_ANTI_CD3_STIM_ELK1_KO_DP_THYMOCYTES_DN
GSE6090_UNSTIM_VS_DC_SIGN_STIM_DC_DN
MODULE_108
GO_MOTOR_NEURON_AXON_GUIDANCE
CDC5_01
GO_CELL_MIGRATION_INVOLVED_IN_SPROUTING_ANGIOGENESIS
CHR11Q12
NFE2L2.V2
GSE11961_FOLLICULAR_BCELL_VS_MEMORY_BCELL_DAY40_DN
GO_REGULATION_OF_MUSCLE_CONTRACTION
GO_POSITIVE_REGULATION_OF_SMOOTH_MUSCLE_CONTRACTION
GO_REGULATION_OF_CHONDROCYTE_DIFFERENTIATION
CHR3Q13
GO_B_CELL_PROLIFERATION_INVOLVED_IN_IMMUNE_RESPONSE
GO_INTERLEUKIN_8_PRODUCTION
GSE40274_FOXP3_VS_FOXP3_AND_HELIOS_TRANSDUCED_ACTIVATED_CD4_TCELL_UP
NF1_Q6_01
GSE28737_WT_VS_BCL6_KO_FOLLICULAR_BCELL_UP
GNF2_IL2RB
GSE29949_MICROGLIA_BRAIN_VS_CD8_POS_DC_SPLEEN_DN

Minor issues:

* Page 2: state-of-the-field should be state-of-the-art
* P 3: diagnose should be diagnoses

* P 3: caring should be care.
* There are many more, the author must revise the grammar again.

A: Thank you. We agree with the reviewer. We have proofread the manuscript thoroughly and now revised our manuscript based on the above suggestions.
